# The cooperative behavioral game of urban waste reduction network considering environmental cost-sharing

**DOI:** 10.1371/journal.pone.0356910

**Published:** 2026-09-01

**Authors:** Xiaoxia Jia, Tingge Wu, Xiaoyue Hu, Weiyi Guang

**Affiliations:** 1 School of Management, University of Shanghai for Science and Technology, Shanghai, China; 2 School of Management, Shanghai University, Shanghai, China; USTC: University of Science and Technology of China, CHINA

## Abstract

This study aims to identify key intervention points for urban solid waste reduction and assign corresponding environmental responsibilities. It further examines how enterprises engage in waste reduction and cooperate with one another in this process. The research integrates Material Flow Analysis (MFA) and Life Cycle Assessment (LCA) to locate critical nodes for waste reduction. An evolutionary game model is developed, where enterprises are modeled as nodes in a scale-free network and interact with neighboring nodes. MATLAB simulations are conducted to evaluate the effects of cost-sharing, benefit allocation, penalty mechanisms, and spillover effects on cooperative behavior in waste reduction. The results reveal that moderate penalty mechanisms and positive spillover effects promote multi-subjects cooperative waste reduction. While cost-sharing and benefit allocation mechanisms also enhance cooperation, their effectiveness depends on maintaining parameter values within reasonable ranges. Furthermore, the impact of these mechanisms varies across networks of different scales, suggesting that a one-size-fits-all approach may be ineffective. This study provides a novel integration of MFA, LCA, and evolutionary game theory within a network framework to explore enterprise-level cooperation in waste reduction. It offers actionable insights into designing differentiated incentive mechanisms based on network characteristics, contributing to more effective urban waste management strategies.

## 0 Introduction

Rapid economic development implies huge inherent demand and the proliferation of municipal solid waste has become a major obstacle to constructing and protecting China’s ecological environment. To cope with the increasing municipal solid waste and seek reasonable disposal solutions, in 2018, UN-Habitat advocated that cities worldwide participate in the ‘Waste Wise Cities’, encouraging all parties to take measures to meet the waste reduction challenge [1]. The construction of ‘waste-free cities’ is an important strategic deployment in China, which is accelerating the achievement of sustainable development and circular economy through the implementation of a variety of policies, while encouraging more subjects to participate in urban waste reduction [2]. Solid waste, from generation, collection, and utilization to disposal, relates to many subjects of interest such as manufacturers, retailers, waste collection companies, waste treatment companies, etc., with intricate links between corporations and a certain degree of dependence between them. With the promotion of waste reduction policies and the participation of more and more corporations, it is of great practical significance to study the waste reduction behaviors among corporations. To sort out the interrelationships of each corporate subject in the process of waste reduction concisely, the concept of network is proposed to be introduced, and the various types of corporate subjects involved in urban waste reduction are abstracted as nodes of the network, and the relationships between corporations are abstractly represented as edges in the network, to build a framework of urban waste reduction network.

Admittedly, the environmental cost is a necessary expense for every enterprise participating in waste reduction in the process of waste reduction [3], and the distribution of environmental cost among the members of the waste reduction network will inevitably become a concern of the network participants, which is directly related to the enthusiasm of the enterprises to participate in waste reduction in the whole process of solid waste from none to none. In other words, unreasonable cost allocation will affect the waste reduction efficiency of the whole network, aggravate the burden of some enterprises, and weaken the willingness of some enterprises to cooperate. Therefore, to promote inter-enterprise cooperation in waste reduction, it is important to pay attention to network member enterprises’ willingness to waste reduction and their environmental responsibility, and on this basis, to explore the continuous game of inter-enterprise cooperation in waste reduction, and to reveal the strategic choices of each subject based on the maximization of their interests in the process of the evolutionary game, which is obviously of great theoretical and practical significance to improve the operational efficiency and effectiveness of urban waste reduction network cooperation. It is of great theoretical value and practical significance to enhance the operational efficiency and effectiveness of multi-subjects urban waste reduction network cooperation.

This study makes several contributions to the literature. Firstly, unlike previous research focusing on estimating a certain stage of urban waste reduction such as waste disposal or its associated participants such as the game between waste collection, transportation enterprises and waste disposal enterprises, the present study investigates all environmentally relevant enterprises throughout the entire product life cycle and takes multiple influencing factors into consideration. By adopting this approach, the study provides novel insights grounded in the frameworks of cooperative network theory. Secondly, the previous research has concentrated on the waste reduction policies targeting government administration side,exploring aspects like tax subsidies and violation penalties. However, limited attention has been assumed to the urban cooperation side influencing environment governance.Conversely, this study scrutinizes key intervention points for urban solid waste reduction and corresponding environmental responsibilities from the cooperation of urban reduction network composed of enterprises participating in all stages. Thirdly, current research adopts a scale-free network to characterize and describe the stability of inter-enterprise cooperative relationships in urban waste reduction. It eliminates the interference of the complete rationality assumption in classical game theory and the relatively static nature of traditional game models, and accurately identifies the behavioral response strategies of each participant in the waste reduction cooperation network.

This study draws corresponding practical implications.Studying the waste reduction cooperative behavior of network nodes can stabilize the cooperative relationship among all nodes and help achieve the goal of urban solid waste minimization. Moreover, taking environmental cost-sharing into account enables a fairer and more reasonable allocation of environmental costs among enterprises, while safeguarding the interests of all participating enterprises.Based on the simulation results, this study intends to put forward targeted suggestions in order to attract more stakeholder enterprises to actively participate in waste reduction cooperation and promote the efficient operation of the waste reduction cooperation network.

## 1 Review of relevant national and international literature

### 1.1 Status of research on environmental cost-sharing

Western countries researched earlier on the environmental cost, Beams and Fertig [4] and Marlin [5] were the first to study environmental cost accounting and became pioneers in the field. Chinese research in environmental cost management began later, Ge and Li mainly introduced the reasons and theoretical methods and so on about the emergence of green accounting in foreign countries and sorted out the international trends in the development of green accounting theory, which triggered a new wave of research by domestic scholars on China’s environmental accounting [6].

With the rapid development of the economy, people’s life needs are becoming richer and richer, and the consumption of the natural environment in the process of daily production and operation is also increasing. In 1994, the Chinese government promulgated China’s Agenda 21, which emphasized the need to use the market mechanism to encourage enterprises to take environmental costs into account in the process of production and operation and decision-making analysis and to gradually change the way of using the environment free of charge and passing on the costs to the society [7]. The document emphasizes the need to use market mechanisms to encourage enterprises to consider environmental costs in their production operations and decision-making analysis and to gradually change the practice of using the environment without compensation and transferring part of the costs to society. Patrick and Francois constructed the Environmental Engineering Group environmental costing model to facilitate environmental costing in South Africa and conducted a case study based on the production process of cigarettes [8]. Mylonakis and Tahinakis propose the use of a cost-benefit analysis model as a methodology to estimate the environmental revenues and costs generated in Greece and to verify the intrinsic mechanism of action between the environment and the economic performance of firms through the use of green accounting information system [9].

To protect the environment more reasonably and efficiently and realize the long-term goal of sustainable development, it is crucial to study the reasonable apportionment of environmental costs among all relevant subjects. Bhaskaran and Krishnan studied the impact of the cost-sharing mechanism on the product design and development of a company by using the Nash bargaining model and verified the results with examples [10]. Kawasaki et al. established a low-carbon supply chain network between three countries, China, Japan, and Malaysia, through discrete-event simulation, and used simulation to analyze the relationship between environmental costs, carbon dioxide emissions, and delivery times in the network [11]. Li et al. explored how quality and pricing decisions in supply chains are affected by Nash bargaining fairness concerns and analyzed the role of fairness concerns on supply chain coordination and profit distribution by constructing a mathematical model [12]. Li et al. investigated how fair allocation mechanisms can enhance cooperation efficiency and environmental sustainability by analyzing low-carbon supply chain strategies based on revenue-sharing and cost-sharing contracts, and the results demonstrated how supply chain members can allocate benefits to optimize the low-carbon transition under contractual coordination [13].

### 1.2 Current status of research in urban waste reduction

Regarding the research on urban waste reduction, scholars at home and abroad mainly analyze and argue from various perspectives, such as punishment mechanisms, benefit distributions, and spillover effects.

#### 1.2.1 Penalty mechanism perspective.

Global production and development consume significant resources and generate substantial waste. Scholars have proposed various mechanisms for waste management. Fullerton and Kinnaman examined mechanisms in the UK involving taxation and subsidies for recycling [14], while Poon noted that many countries have adopted measures such as tax incentives, subsidies, and penalties to address the surge in waste [15]. However, in practice, the effectiveness of these policies has been limited, mainly due to a lack of systematic analysis of decision-making motivations from a multi-stakeholder perspective. To enhance waste management efficiency, Wang et al. recommended setting recycling rate targets to assess industry performance [16]. Peng et al. used a game model to reveal that enterprises’ environmental responsibility is directly influenced by government supervision and penalties, and reasonable punishment mechanisms can significantly improve corporate environmental performance [17]. Wang and Shi compared static and dynamic punishment mechanisms, finding that dynamic mechanisms more effectively and consistently drive industrial enterprises to reduce pollution [18]. Moreover, Chen and Ulya constructed a green supply chain model, demonstrating that reward and punishment mechanisms can improve the overall recycling rate [19]. Du et al. analyzed interactions among governments, contractors, and consumers, using simulations to define reasonable ranges for incentives and penalties to optimize construction waste management [20]. Rathore and Sarmah found that reward and punishment mechanisms significantly improved the efficiency of municipal solid waste supply chains, outperforming simple subsidies. These studies highlight the necessity of designing incentive and penalty mechanisms from a multi-stakeholder perspective to improve waste management outcomes [21].

#### 1.2.2 Benefit distribution perspective.

Fudenberg and Tirole emphasize the importance of game models in explaining resource allocation and benefit sharing in cooperative games, and through a game-theoretic framework, they analyze how long-term cooperative relationships and strategic equilibria between corporations can be affected when they adopt different mechanisms for distributing benefits [22]. Cvitanic used real options theory to simulate the decision-making behavior of an R&D cooperation alliance formed by two firms providing new product development and R&D resources and found that the profit distribution between the two firms is optimal when a linear sharing rule is used [23]. Gromova does this by exploring the application of shapely values in dynamic games, in particular how to distribute gains in an ongoing cooperative environment to ensure long-term cooperation and benefit-sharing among the various players [24]. Panico analyses the evolutionary process of collaboration in strategic alliances, in particular the impact of benefit distribution and trust on the stability of long-term cooperation. The article examines the evolutionary patterns and impact of collaboration from a dynamic perspective [25]. Zhou et al. combined the product collaborative innovation cooperation network with evolutionary game theory, constructed a game model based on the influencing factors of benefit distribution, and analyzed the cooperative behaviors of each node through simulation, and the results showed that adding appropriate weights and distribution coefficients when distributing benefits can make the main corporations in the cooperation network obtain higher benefits, and at the same time, prevent the profits of the micro and small corporations from being damaged [26].

#### 1.2.3 Spillover effects perspective.

macdougall is a pioneer in the field of spillovers and was the first to carry out systematic analysis and research on them [27]. d’aspremont and jacquemin constructed a two-stage double oligopoly model and studied the research and development spillovers in the model, laying a foundation for scholars to study the development of cooperation among enterprises [28]. meagher and rogers studied the spillover effect among enterprises in innovation cooperation network, and the research results showed that the innovation spillover effect is related to the density of cooperation network among enterprises, the ability difference of different enterprises, etc., and the structure and function of the network also affect the innovation spillover effect of enterprises, which in turn affects the innovation rate of the whole cooperation network [29]. huber studied the effect of knowledge spillover in enterprise clusters and found that knowledge spillover can increase the knowledge stock of the whole cluster, which can improve the innovation motivation of enterprises and increase their competitive advantage in the industry [30]. tang et al. used the entropy weight topsis method to construct a multilayer index to measure the environmental governance level index of 30 provinces, and at the same time constructed a spatial durbin panel model to further study the influence of spatial spillover effects on the environmental governance level [31].

### 1.3 Review of the current state of research

After combing the literature on environmental cost sharing, it is found that environmental costs were initially applied to the accounting of individual enterprises, and with the continuous communication and cooperation among enterprises, scholars gradually began to study the sharing of environmental costs among enterprises, and most scholars conducted research based on a certain type of enterprises or certain enterprises in the supply chain, while few scholars conducted research based on the perspective of cooperative network on the sharing of environmental costs. Few scholars have studied the environmental cost-sharing problem based on the perspective of cooperative networks. Moreover, the environmental cost-sharing factor is mostly used to study cooperative R&D and low-carbon cooperation among enterprises, and few scholars have used the environmental cost-sharing factor to study the cooperative behavior of urban waste reduction.

The articles in the research field of cooperative behavior of urban waste reduction are sorted out and classified based on different perspectives. At present, most of the relevant studies on urban waste reduction start from a certain link or the subjects involved in a certain link, such as: constructing the game model between residents and the government in the segment of domestic garbage classification, constructing the game model between manufacturers and retailers in the segment of production and sales, and constructing the game model between garbage collection enterprises and garbage disposal enterprises in the stage of waste disposal. The game model between garbage collection enterprises and garbage disposal enterprises in the waste disposal stage. Moreover, many scholars only start from a certain perspective to study the influence of individual influencing factors on the cooperative behavior between subjects, and few scholars have comprehensively researched the enterprises involved in all aspects of the whole life cycle of the product and considered a variety of influencing factors.

Therefore, this paper will start from the perspective of inter-enterprise waste reduction network cooperation, firstly, identify the network nodes and analyze the characteristics of the node enterprises based on the material flow and Life Cycle Assessment, then construct the scale-free waste reduction network cooperation and analyze the characteristics of the network, and then put forward the basic assumptions based on the influencing factors and set up the evolution game model on the network based on this. Secondly, the evolutionary game path of waste reduction cooperation is calculated by copying the dynamic equations, the equilibrium point is found according to the local stability of the Jacobi matrix, and the evolutionary stabilization strategy of the enterprise waste reduction cooperation is discussed according to the equilibrium point in the scenarios to provide a theoretical basis for the simulation analysis. Finally, MATLAB software to simulate and analyze the evolutionary stabilization scenarios of the cooperative network and to study the cooperative behaviors of enterprises by integrating various influencing factors.

## 2 Problem description and model assumptions

### 2.1 Reduction nodes of solid waste based on material flow and life cycle assessment integration with the definition of environmental responsibility

Material flow analysis generally refers to the systematic analysis of material flow and storage in a specific system within a certain spatial and temporal scope and is a tool for studying the trajectory of material resources in economic production activities. Life Cycle Assessment, or the principle of product life cycle assessment, is an environmental management tool that is often used to analyze the resources consumed in the whole process of production of a certain product as well as define the responsibility for environmental impacts. Combining material flow and Life Cycle Assessment to study solid waste management can not only make up for the disadvantage of material flow, which only considers material flow but lacks information on environmental impact, but also define the environmental responsibility of the main body from the aspects of material flow and environmental impact.

**Fig 1:**
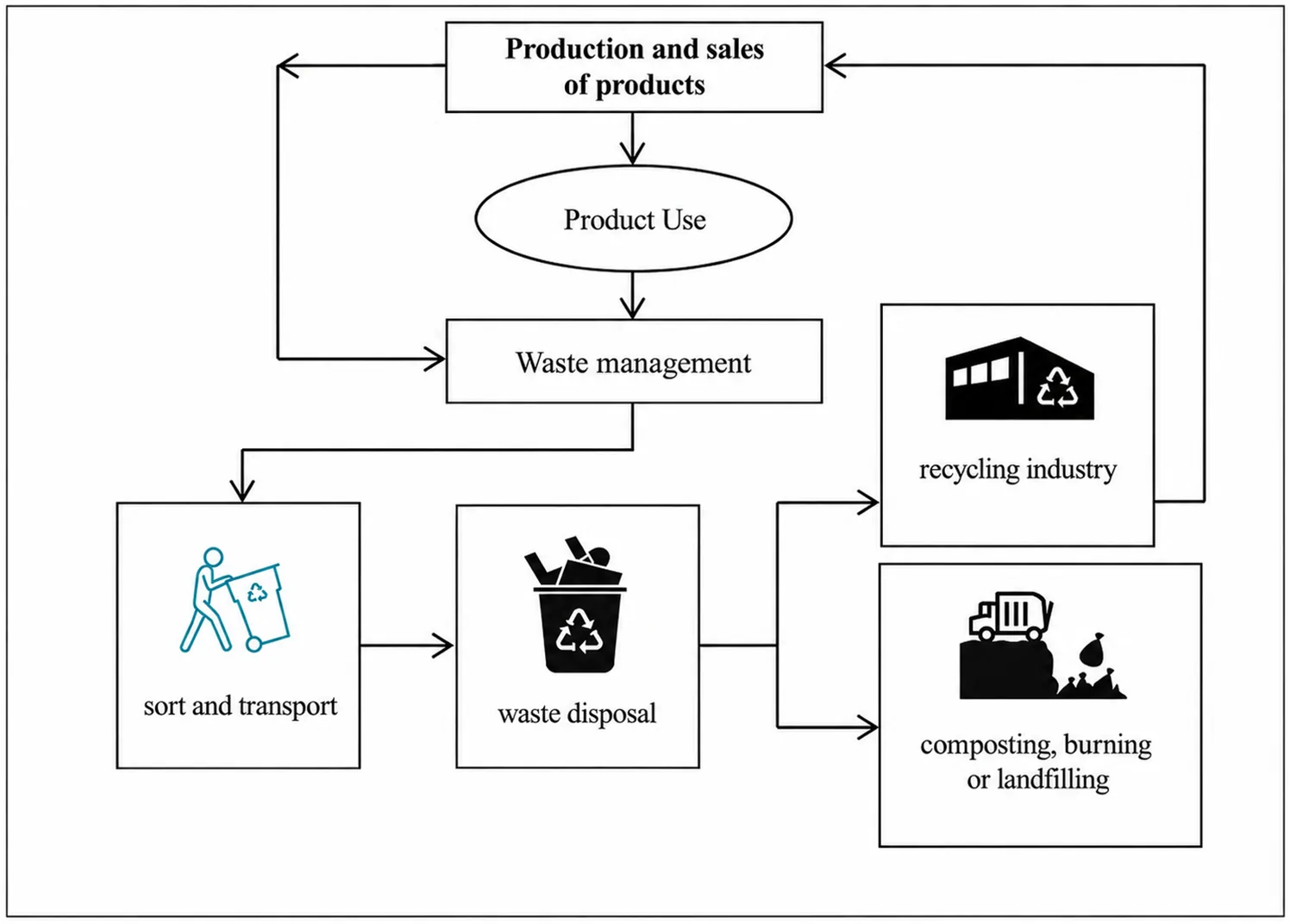
Diagram of total life cycle management of solid waste incorporating material flows.

Solid waste generally includes domestic garbage, construction waste, and waste electrical and electronic products, etc. Promoting the management of domestic garbage is related to people’s livelihood and public interest, and is an important project of social governance. This paper mainly focuses on solid waste as a material flow to carry out research and analyze the life cycle of solid waste, the flow trajectory of domestic waste in the whole life cycle of solid waste is shown in Fig 1. Solid waste minimization involves multiple nodes. In the stage of product production and sales, manufacturing enterprises will produce two parts of waste in the process, one part is the waste generated in the production process, and the other part is incidental to the product flow to the next stage, such as the packaging of the product, the packaging used in the process of transportation and sales, and so on. The product use stage mainly involves consumer participation, but this article is based on a reduction study from a business perspective, so this stage is not considered. The waste management stage mainly includes the classification, transportation, and disposal of garbage, and the reasonable classification of domestic garbage is an important measure of quantitative reduction, which can separate the valuable parts of garbage to realize regeneration and utilization and reduce the amount of harmless disposal of garbage. Waste disposal is also divided into two parts: the resource waste is transported to renewable resource recycling enterprises for specialized disposal and regeneration, and ultimately flows to manufacturing enterprises for recycling, while the remaining non-renewable portion can be transported to waste incineration or composting enterprises for harmless treatment.

Obviously, the solid waste minimization node mainly includes the product production and marketing stage, the waste separation and transportation stage, and the waste disposal stage. The product production and sales stage has the responsibility of reducing the waste generated during the production process and reducing the flow of hidden waste such as packaging to the next stage; the waste separation and transportation stage has the responsibility of rationally classifying garbage and separating renewable and recyclable waste; and the waste disposal stage has the responsibility of increasing the rate of harmless treatment of garbage and improving the recycling rate of recyclable waste.

### 2.2 Definition and construction of inter-enterprise waste reduction network nodes considering environmental cost-sharing

#### 2.2.1 Network setup.

A waste reduction cooperation network is a network structure established by waste reduction enterprises for common waste reduction goals, aimed at strengthening waste reduction cooperation among enterprises and improving the enthusiasm for waste reduction cooperation among enterprise subjects. Inter-enterprise waste reduction cooperative behavior network is similar to the structural characteristics of a scale-free network, which is mainly reflected in the following aspects: firstly, there are enterprises with higher influence and industry status in waste reduction enterprises, such as large-scale production and sales enterprises, which play a core role in the whole cooperative network, but the number of these enterprises is relatively small, and most of the nodes have a relatively low degree of enterprises, which is in line with the characteristics of the power law distribution of scale-free network; secondly, the inter-enterprise waste reduction cooperative behavior network has been established for common waste reduction goals. Second, the cooperation between enterprises in the waste reduction network is constantly updated, and the selection strategy of each node in the network is not the same, some node enterprises will choose to cooperate, while others are reluctant to cooperate. With the increasing number of rounds of the game, the selection strategy of the node enterprises is also constantly changing, and the node enterprises are constantly changing the object of cooperation in waste reduction. Thirdly, enterprises in the waste reduction cooperation will give priority to enterprises with strong strength, waste reduction technology, and the ability to cooperate, so the connection between nodes in the waste reduction network cooperation is optimal rather than random, which coincides with the characteristics of the optimal connection of the scale-free network.

The number of enterprises involved in the network of waste reduction cooperation behavior plays an important role in the evolution of the network, and the more subjects involved in waste reduction cooperation represent the larger network size. To facilitate the study of the impact of the number of waste reduction participating subjects on the evolution of the cooperative network, this paper refers to the practice of Li et al., and sets the network size as 50 nodes, 100 nodes, and 500 nodes of the network, respectively [32].

#### 2.2.2 Model hypothesis.

Based on the structural characteristics of the cooperative network constructed by the waste reduction behaviors of the enterprise subjects, and combined with the actual scenarios, the following four hypotheses are proposed:

Hypothesis 1: Inter-enterprise waste reduction network cooperation is denoted as G=(V,E) whereV denotes the set of all network nodes in the waste reduction network cooperation, and  E denotes the set of all edges connecting the nodes in the waste reduction network cooperation.

Hypothesis 2: When each enterprise chooses the game opponent, due to the limited access to information, an enterprise cannot communicate and establish contact with every enterprise in the network, and the enterprises in the actual scenario generally choose the neighboring enterprises to play the game.

Hypothesis 3: Enterprises in the cooperative network are finitely rational, the probability of a firm choosing a waste reduction cooperative strategy is related to the amount of gain, and there is also the possibility of not choosing the optimal strategy due to misinformation.

Hypothesis 4: When each participating enterprise follows a certain updating principle and makes a choice of strategy with a certain probability, depending on the results of the previous round of the game, the gains obtained by the adoption of the waste reduction strategy are derived from the gains of waste reduction cooperation.

#### 2.2.3 Network update rules.

By constructing a scale-free network model, each waste reduction enterprise represents a node in the network, and the connection and interaction between enterprises are expressed as the connecting edges between nodes, and the nodes can choose “cooperation” and “non-cooperation” strategies in the process of the game after initializing the cooperation of the waste reduction network, and the nodes’ choice of strategy is constantly updated and changed in the process of the game. Assuming that a node enterprise is represented by *i* in the cooperative network and the neighboring enterprises of enterprise *i* are represented by *j*. At the end of each round of the game, the enterprises will compare each other’s returns, and when the average expected return obtained by enterprise *i* at the time t is smaller than that obtained by the neighboring enterprises, it will change its strategy, imitate and learn the strategy of the neighboring enterprise *j*. On the contrary, when the expected return obtained by enterprise *i* is higher than that of the neighboring enterprise *j*, its strategy will remain unchanged.

The probability that enterprise *i* chooses a waste reduction cooperative strategy at moment t in a complex network of firms’ waste reduction cooperative behavior is$\;P_{i}(t)$ enterprise *i* plays a game with neighboring enterprises with probability${\;P}_{i}(t)$ and neighboring enterprises to play the game, after each round of the game, the game participants will compare the gains to the neighboring participants, when it is found that the neighboring participants of the gain are greater than their own gain obtained, they will learn and imitate the neighboring participants of the strategy, the neighboring enterprise’s strategy into their own strategy and this strategy as the next round of the game strategy. When enterprise *i* does not reach a steady state at the moment *t*, at the moment *t + 1*, it imitates and learns wi*t*h probability W using the Fermi function as the update rule, and again at the moment *t + 1* when it does not reach stabilization, it will be updated with *W* probability at the moment *t + 2* with $V_{ij}$ probability of disconnecting and reconnecting with other enterprise nodes in the waste reduction network cooperation, and then repeat the above update rule until reaching the stable state.

### 2.3 Study on the structural characteristics of inter-enterprise waste reduction network cooperation considering environmental cost-sharing

The enterprises involved in waste reduction are distributed in various industries in the north and south of the Yangtze River, although the space and other factors will make the cooperation between enterprises restricted and affected, the enterprises can be constantly exchanged and contacted through the flow of materials and funds. Therefore, the main body of each enterprise can be abstracted and simplified as the nodes in the network, and the relationship and connection between enterprises can be abstracted and simplified as the connecting edges between the nodes in the network. Inter-enterprise waste reduction network cooperation has the following characteristics:

#### 2.3.1 Uneven distribution of degree value nodes.

Inter-enterprise waste reduction network cooperation is not only to carry out waste reduction exchanges between the relevant enterprises but also to carry out exchanges and contacts with other subjects due to legal, policy, and technical factors. In the cooperation network, the more edges a node has, the more enterprises have contact with the node, the higher the degree of the node, and the richer the resources and information the node has. But enterprises with full resources and information will only account for a small proportion, and the majority of enterprises can only grasp a small part of the resources and information, so the high value of the node is relatively small while the low value of the node is relatively more, Enterprises with insufficient information resources need to rely on the node of the higher value of the node enterprise to draw more information and resources.

#### 2.3.2 High clustering coefficient.

The inter-enterprise solid waste reduction cooperation based on the combination of material flow and Life Cycle Assessment presents the development characteristics of integrated waste reduction, which can promote waste reduction cooperation and information sharing among enterprises, and at the same time, the clustering coefficient of inter-enterprise waste reduction cooperation is improved. For example, if the producer establishes commodity supply cooperation with distributor A and distributor B at the same time, then the producer can guide distributor A and distributor B to establish some contacts, which will increase the clustering coefficient between the enterprises The higher the clustering coefficient, the closer the links between neighboring enterprises will be, the more efficient the transmission of waste reduction information between enterprises will be, which can actively promote the cooperative behavior of waste reduction between enterprises.

#### 2.3.3 Shorter average paths.

The average path is usually used in cooperative networks to measure the average distance between node firms, which in inter-firm waste reduction network cooperation corresponds to the average number of firms involved in the cooperation. In waste reduction network cooperation, if the average path is shorter, then the communication speed between node enterprises will be faster. A shorter average path will make the information transmission speed between nodes faster, and will also enable the node enterprises in the network to use less time to obtain more information on waste reduction and promote the transmission of funds and technology, etc. in the network.

## 3 Model construction

### 3.1 Basic hypothesis

Based on the cooperation of the urban waste reduction network, we construct the game model of waste reduction cooperation behavior between network nodes and neighboring nodes from the perspective of waste reduction behavior among enterprises within the cooperation network. The evolution of the cooperative network is a process of continuous gaming between each node on the network and its neighboring nodes, so to make the study clearer and more concise, we set the game subject as enterprise i and neighboring enterprise j, i.e., a node in the network and its neighboring nodes. It is assumed that each node will fully consider its interests when acting, and each node has limited rationality, before the node carries out the game and cannot accurately obtain the neighboring node enterprise’s revenue and strategy information, each node can learn from each other to continuously improve their strategies, until the reduction of waste network cooperation to reach a stable state.

The choice of action strategies for enterprise i and neighboring enterprise j is {cooperation, no cooperation}. Cooperation refers to the process in which participating enterprises work together for the common goal of waste reduction and potential benefits, in which the participating enterprises give full play to their resource advantages and cooperate with other enterprises in the game, in the process of improving their competitive advantages, increasing the enterprise’s revenue, and maximizing their benefits. Non-cooperation means that one of the two parties to the game cooperates with the other party in the process of realizing the goal of waste reduction with a negative attitude, without paying any cost for it and without paying any effort for it but still can get some benefits from it, but need to bear a certain amount of penalties for breach of contract. According to the influencing factors of enterprise waste reduction cooperation, we make the following assumptions for the establishment of the municipal waste reduction cooperation game model:

**Hypothesis 1:** Both enterprises will inevitably have to invest some costs in the process of waste reduction cooperation to realize the goal of waste reduction. The cost invested by enterprise *i* in the waste reduction cooperation is denoted by $c_{i}$ The cost invested by enterprise *i* in the waste reduction cooperation is denoted by $c_{j}$ is the cost invested by the two enterprises in the process of cooperation.$c_{i}+c_{j}$ is the total cost invested by the two enterprises in the cooperation process. Two subjects in the process of cooperation and input also need to take into account the impact of cost-sharing factors, the two sides *i* by a certain proportion of the cost of sharing, enterprise *i* and enterprise j cost-sharing coefficients were used as$\;\alpha_{i}$ and${\;\alpha }_{j}$ respectively, and$\;\alpha_{i}+\alpha_{j}=\text{1}.$ Therefore, the cost shared by enterprise i in the waste reduction cooperation can be expressed as $\alpha_{i}\left(c_{i}+c_{j}\right)$. The cost shared by enterprise j in the waste reduction cooperation can be expressed as $\alpha_{j}\left(c_{i}+c_{j}\right)$.

**Hypothesis 2:** The two enterprises will generate corresponding cooperative benefits in the process of cooperation, and the benefits of waste reduction inputs from enterprise *i* and enterprise *j* are respectively$\;r_{i}$ and $r_{j}$ At the same time, we also need to consider the impact of the benefit distribution factors, when the two companies signed a waste reduction cooperation agreement, the contract will be agreed in advance on the proportion of their respective revenue sharing, enterprise *i* and enterprise *j* benefit distribution coefficients are used, respectively $\beta_{i}$ and $\beta_{j}$ and $\beta_{i}+\beta_{j}=\text{1}$. Therefore, the benefit distribution of enterprise *i* in the cooperative behavior of waste reduction is expressed as$\;\beta_{i}\left(r_{i}+r_{j}\right).$ The benefit distribution of enterprise *j* in the cooperative behavior of waste reduction is denoted as${\;\beta }_{j}\left(r_{i}+r_{j}\right)$.

**Hypothesis 3:** In the process of waste reduction cooperation, if either party shifts its strategy or finds that the benefits are not substantial and not satisfactory, or believes that it can achieve the waste reduction goal alone, it will choose to violate the waste reduction cooperation agreement and act alone, and then the defaulting enterprise will need to bear the responsibility for the breach of contract, and part of the breach of contract penalties are used to make up for the other party’s loss of cooperation. The penalty for violation of the waste reduction cooperation agreement by either party is denoted byδ is denoted by δ>0.  The penalty for a violation of a waste reduction cooperation agreement by either party is denoted by.

**Hypothesis 4:** In the process of waste reduction cooperation, it is assumed that enterprise *j* will generate certain external benefits, due to the existence of waste reduction information sharing and complementarity, even if enterprise *i* does not pay any extra cost can also obtain part of the waste reduction benefits of enterprise *j*. The waste reduction spillover effect of enterprise *i* is denoted by${\;\mu }_{i}\gamma_{i}r_{j}$ where $\mu_{i}$ is the coefficient of waste reduction spillover effect of enterprise *j* on enterprise *i* in cooperation, and ${\;\gamma }_{i}$ denotes the waste reduction resource benefit that exists in enterprise i itself.

**Hypothesis 5:** At the beginning of the evolutionary game, the probability that enterprise i chooses “cooperation” is x, and the probability that enterprise j chooses “cooperation” is y, and the probability that enterprise j chooses “non-cooperation” is (1-y). The probability of enterprise j choosing “cooperation” is y, and the probability of choosing “non-cooperation” is (1-y).

Based on the above assumptions, the game benefit matrix for municipal waste reduction cooperation is constructed as shown in Table 1:

**Table 1:** Municipal waste reduction cooperative behavior game benefits matrix.

Corporation 𝐢	Enterprise *j*	
	collaborative	uncooperative
collaborative	$\beta_{i}\left(r_{i}+r_{j}\right)+\mu_{i}\gamma_{i}r_{j}-\alpha_{i}\left(c_{i}+c_{j}\right)$ $\beta_{j}\left(r_{i}+r_{j}\right)+\mu_{j}\gamma_{j}r_{i}-\alpha_{j}\left(c_{i}+c_{j}\right)$	$\delta -\alpha_{i}\left(c_{i}+c_{j}\right)$ $\mu_{j}\gamma_{j}r_{i}-\delta$
uncooperative	$\mu_{i}\gamma_{i}r_{j}-\delta$ $\delta -\alpha_{j}\left(c_{i}+c_{j}\right)$	0 0

### 3.2 Modeling

The nodes in the waste reduction network cooperation are all limited rational game subjects, the nodes cannot choose the optimal action strategy with only one choice, the nodes continuously learn from other nodes in the waste reduction network cooperation and communicate with each other, and then optimize their own action strategy, so as to achieve a stable state.

The game payoff matrix of inter-firm cooperative behavior for waste reduction is solved according to Table 1 and Hypothesis 5.

The expected benefit to enterprise *i* of adopting a “cooperative” strategy is:


$$\begin{array}{c} {\text{U}}_{\text{1}}^{\text{1}}=\text{y}\left[\beta_{\text{i}}\left({\text{r}}_{\text{i}}+{\text{r}}_{\text{j}}\right)+\mu_{\text{i}}\gamma_{\text{i}}{\text{r}}_{\text{j}}-\alpha_{\text{i}}\left({\text{c}}_{\text{i}}+{\text{c}}_{\text{j}}\right)\right]+(\text{1}-\text{y})\left[\delta -\alpha_{\text{i}}\left({\text{c}}_{\text{i}}+{\text{c}}_{\text{j}}\right)\right] \end{array}$$ (1)

The expected benefit to enterprise *i* of adopting a “non-cooperative” strategy is:


$$\begin{array}{c} {\text{U}}_{\text{1}}^{\text{2}}=\text{y}\left(\mu_{\text{i}}\gamma_{\text{i}}{\text{r}}_{\text{j}}-\delta \right)+(\text{1}-\text{y})\text{0}=\text{y}\mu_{\text{i}}\gamma_{\text{i}}{\text{r}}_{\text{j}}-\text{y}\delta \end{array}$$ (2)

From equations (4), the expected return of firm i adopting a mixed strategy is:


$$\begin{array}{c} U_{\text{1}}=xU_{\text{1}}^{\text{1}}+(\text{1}-x)U_{\text{1}}^{\text{2}} \end{array}$$

$$\begin{array}{c} =\text{xy}\beta_{i}\left(r_{i}+r_{j}\right)+y\mu_{i}\gamma_{i}r_{j}-x\alpha_{i}\left(c_{i}+c_{j}\right)-y\delta +x\delta \end{array}$$ (3)

Based on the expected returns of firm i under the above strategies, the replication dynamics equation for enterprise *i* adopting the “cooperative” strategy can be derived as follows:


$$\begin{array}{c} \text{F}(x)=\frac{dx}{dt}=x\left(U_{\text{1}}^{\text{1}}-U_{\text{1}}\right)=x(\text{1}-x)\left(U_{\text{1}}^{\text{1}}-U_{\text{1}}^{\text{2}}\right)\; \end{array}$$

$$\begin{array}{c} =x(\text{1}-x)\left[y\beta_{i}\left(r_{i}+r_{j}\right)+\delta -\alpha_{i}\left(c_{i}+c_{j}\right)\right] \end{array}$$ (4)

Similarly, the expected payoff to firm j from adopting the “cooperation” strategy is:


$$\begin{array}{c} U_{\text{2}}^{\text{1}}=x\left[\beta_{j}\left(r_{i}+r_{j}\right)+\mu_{j}\gamma_{j}r_{i}-\alpha_{j}\left(c_{i}+c_{j}\right)\right]+(\text{1}-x)\left[\delta -\alpha_{j}\left(c_{i}+c_{j}\right)\right] \end{array}$$ (5)

The expected payoff to firm j for adopting a “non-cooperative” strategy is:


$$\begin{array}{c} {\text{U}}_{\text{2}}^{\text{2}}=\text{x}\left(\mu_{\text{j}}\gamma_{\text{j}}{\text{r}}_{\text{i}}-\delta \right)+(\text{1}-\text{x})\text{0}=\text{x}\mu_{\text{j}}\gamma_{\text{j}}{\text{r}}_{\text{i}}-\text{x}\delta \end{array}$$ (6)

From equations (5) and (6), the expected return for firm j to adopt a mixed strategy is:


$$\begin{array}{c} U_{\text{2}}=yU_{\text{2}}^{\text{1}}+(\text{1}-y)U_{\text{2}}^{\text{2}} \end{array}$$

$$\begin{array}{c} \;=\text{yx}\beta_{j}\left(r_{i}+r_{j}\right)+x\mu_{j}\gamma_{j}r_{r}-y\alpha_{j}\left(c_{i}+c_{j}\right)-x\delta +y\delta \end{array}$$ (7)

Based on the expected returns of enterprise *j* under the above strategies, the replication dynamics equation for firm j adopting the “cooperative” strategy can be derived as follows:


$$\begin{array}{c} \text{F}(y)=\frac{dy}{dt}=y\left(U_{\text{2}}^{\text{1}}-U_{\text{2}}\right)=y(\text{1}-y)\left(U_{\text{2}}^{\text{1}}-U_{\text{2}}^{\text{2}}\right)\; \end{array}$$

$$\begin{array}{c} \;=y(\text{1}-y)\left[x\beta_{j}\left(r_{i}+r_{j}\right)+\delta -\alpha_{j}\left(c_{i}+c_{j}\right)\right] \end{array}$$ (8)

If the equilibrium point is required, it is necessary to satisfy  F(x)=0, and F(y)=0, from Eq. (4) and Eq. (8) system of joint equations, let:


$$\begin{array}{c} \left\{ \begin{array}{c} \text{F}(x)=\frac{dx}{dt}=x(\text{1}-x)\left[y\beta_{i}\left(r_{i}+r_{j}\right)+\delta -\alpha_{i}\left(c_{i}+c_{j}\right)\right]=\text{0}\\ \text{F}(y)=\frac{dy}{dt}=y(\text{1}-y)\left[x\beta_{j}\left(r_{i}+r_{j}\right)+\delta -\alpha_{j}\left(c_{i}+c_{j}\right)\right]=\text{0} \end{array}\right. \end{array}$$

Five equilibrium points for the replicator dynamic equations can be derived as${\text{ E}}_{\text{1}}(\text{0},\text{0})$ and${\text{ E}}_{\text{2}}(\text{0},\text{1})$,${\text{E}}_{\text{3}}(\text{1},\text{0})$, ${\text{E}}_{\text{4}}(\text{1},\text{1})$, and${\text{ E}}_{\text{5}}\left({\text{x}}^{*},{\text{y}}^{*}\right)$, where:


$$\begin{array}{c} x^{*}=\frac{\alpha_{j}\left(c_{i}+c_{j}\right)-\delta }{\beta_{j}\left(r_{i}+r_{j}\right)}\;y^{*}=\frac{\alpha_{i}\left(c_{i}+c_{j}\right)-\delta }{\beta_{i}\left(r_{i}+r_{j}\right)} \end{array}$$

### 3.3 Evolutionary stability analysis

#### 3.3.1 Sub-scenario discussion of equilibrium point stability.

To deeply analyze the stabilization strategy of the game system, the Jacobian matrix is constructed by the partial derivatives of the replicator dynamic equations, and the evolutionary stability of the five equilibrium points can be judged by analyzing the local stability of the Jacobian matrix.

From Eq. (4) and Eq. (8), the system Jacobi matrix J can be expressed as follows:


$$\begin{array}{c} \left[\begin{array}{cc} (\text{1}-\text{2}x)\left[y\beta_{i}\left(r_{i}+r_{j}\right)+\delta -\alpha_{i}\left(c_{i}+c_{j}\right)\right] & x(\text{1}-x)\left[\beta_{i}\left(r_{i}+r_{j}\right)\right]\\ y(\text{1}-y)\left[\beta_{j}\left(r_{i}+r_{j}\right)\right] & (\text{1}-\text{2}y)\left[x\beta_{j}\left(r_{i}+r_{j}\right)+\delta -\alpha_{j}\left(c_{i}+c_{j}\right)\right] \end{array}\right] \end{array}$$ (9)

When the Jacobi matrix satisfies the condition of Det(J)>0 and Tr(J)<0, the equilibrium is stable. The evolutionary stabilization strategies for enterprise *i* and *j* can be drawn from$\text{ Det}(\text{J})={\text{a}}_{\text{11}}{\text{a}}_{\text{22}}-{\text{a}}_{\text{12}}{\text{a}}_{\text{21}}$and $\text{Tr}(\text{J})={\text{a}}_{\text{11}}+{\text{a}}_{\text{12}}$. The determinant values and trace values for each equilibrium point of the Jacobi matrix by calculating Det(J) and Tr(J) are shown in Table 2.

**Table 2:** Determinant values and trace values for each equilibrium point of the Jacobi matrix.

Balance point	Det(J)	Tr(J)
$E_{\text{1}}(\text{0},\text{0})$	$\left[\delta -\alpha_{\text{i}}\left({\text{c}}_{\text{i}}+{\text{c}}_{\text{j}}\right)\right]$ $\times \left[\delta -\alpha_{\text{j}}\left({\text{c}}_{\text{i}}+{\text{c}}_{\text{j}}\right)\right]$	$\left[\delta -\alpha_{i}\left(c_{i}+c_{j}\right)\right]$ $+\left[\delta -\alpha_{j}\left(c_{i}+c_{j}\right)\right]$
$E_{\text{2}}(\text{0},\text{1})$	$\left[\beta_{\text{i}}\left({\text{r}}_{\text{i}}+{\text{r}}_{\text{j}}\right)+\delta -\alpha_{\text{i}}\left({\text{c}}_{\text{i}}+{\text{c}}_{\text{j}}\right)\right]$ $\times \left[\alpha_{\text{j}}\left({\text{c}}_{\text{i}}+{\text{c}}_{\text{j}}\right)-\delta \right]$	$\left[\beta_{\text{i}}\left({\text{r}}_{\text{i}}+{\text{r}}_{\text{j}}\right)+\delta -\alpha_{\text{i}}\left({\text{c}}_{\text{i}}+{\text{c}}_{\text{j}}\right)\right]$ $+\left[\alpha_{\text{j}}\left({\text{c}}_{\text{i}}+{\text{c}}_{\text{j}}\right)-\delta \right]$
$E_{\text{3}}(\text{1},\text{0})$	$\left[\alpha_{\text{i}}\left({\text{c}}_{\text{i}}+{\text{c}}_{\text{j}}\right)-\delta \right]$ $\times \left[\beta_{\text{j}}\left({\text{r}}_{\text{i}}+{\text{r}}_{\text{j}}\right)+\delta -\alpha_{\text{j}}\left({\text{c}}_{\text{i}}+{\text{c}}_{\text{j}}\right)\right]$	$\left[\alpha_{\text{i}}\left({\text{c}}_{\text{i}}+{\text{c}}_{\text{j}}\right)-\delta \right]$ $+\left[\beta_{\text{j}}\left({\text{r}}_{\text{i}}+{\text{r}}_{\text{j}}\right)+\delta -\alpha_{\text{j}}\left({\text{c}}_{\text{i}}+{\text{c}}_{\text{j}}\right)\right]$
$E_{\text{4}}(\text{1},\text{1})$	$\left[\alpha_{i}\left(c_{i}+c_{j}\right)-\beta_{i}\left({\text{r}}_{\text{i}}+{\text{r}}_{\text{j}}\right)-\delta \right]$ $\times \left[\alpha_{j}\left(c_{i}+c_{j}\right)-\beta_{j}\left({\text{r}}_{\text{i}}+{\text{r}}_{\text{j}}\right)-\delta \right]$	$\left[\alpha_{i}\left(c_{i}+c_{j}\right)-\beta_{i}\left({\text{r}}_{\text{i}}+{\text{r}}_{\text{j}}\right)-\delta \right]$ $+\left[\alpha_{j}\left(c_{i}+c_{j}\right)-\beta_{j}\left({\text{r}}_{\text{i}}+{\text{r}}_{\text{j}}\right)-\delta \right]$

Since the trace value of the equilibrium point ${\text{ E}}_{\text{5}}\left({\text{x}}^{*},{\text{y}}^{*}\right)$ is at  Tr(J)=0, it does not meet the condition of stability point  Tr(J)<0, ${\text{E}}_{\text{5}}\left({\text{x}}^{*},{\text{y}}^{*}\right)$ must not be an evolutionary system stable point, so only the stability of the other four equilibrium points is analyzed and discussed below.

From the game payoff matrix of urban waste reduction cooperative behavior:$\delta -\alpha_{\text{i}}\left({\text{c}}_{\text{i}}+{\text{c}}_{\text{j}}\right)$ is the difference between the payoffs of firm i choosing the two strategies of cooperation and non-cooperation under firm j adopting the non-cooperation strategy;$\delta -\alpha_{\text{j}}\left({\text{c}}_{\text{i}}+{\text{c}}_{\text{j}}\right)$ is the difference between the benefits of choosing cooperation and non-cooperation for firm j under the non-cooperation strategy of firm i;$\beta_{\text{i}}\left({\text{r}}_{\text{i}}+{\text{r}}_{\text{j}}\right)+\delta -\alpha_{\text{i}}\left({\text{c}}_{\text{i}}+{\text{c}}_{\text{j}}\right)$ is the difference between the returns to firm i’s choice of two strategies of cooperation and non-cooperation under enterprise *j* ‘s adoption of a cooperative strategy; $\beta_{\text{j}}\left({\text{r}}_{\text{i}}+{\text{r}}_{\text{j}}\right)+\delta -\alpha_{\text{j}}\left({\text{c}}_{\text{i}}+{\text{c}}_{\text{j}}\right)$ is the difference between the returns to enterprise *j* ‘s choice of two strategies of cooperation versus non-cooperation under firm i’s adoption of a cooperative strategy.

**Fig 2:**
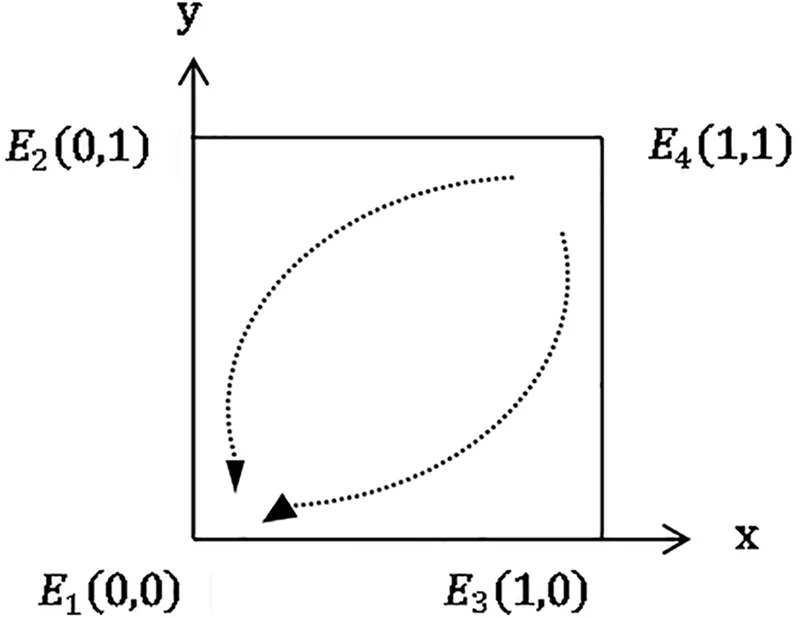
Phase diagram of evolutionary paths-Scenario 1.

According to the sign of the determinant value and trace value under each equilibrium point in the Jacobi matrix, we can analyze whether each equilibrium point is in a stable state, and then further analyze the stabilization strategy of the waste reduction cooperation of each enterprise subject. Here, the stability of each equilibrium point can be discussed in scenarios by the difference of the enterprise’s returns under various scenarios, which are divided into the following six scenarios:

(1) Scenario 1:

When$\left\{ \begin{array}{c} \delta -\alpha_{\text{i}}\left({\text{c}}_{\text{i}}+{\text{c}}_{\text{j}}\right)<\text{0}\\ \delta -\alpha_{\text{j}}\left({\text{c}}_{\text{i}}+{\text{c}}_{\text{j}}\right)<\text{0}\\ \beta_{\text{i}}\left({\text{r}}_{\text{i}}+{\text{r}}_{\text{j}}\right)+\delta -\alpha_{\text{i}}\left({\text{c}}_{\text{i}}+{\text{c}}_{\text{j}}\right)<\text{0}\\ \beta_{\text{j}}\left({\text{r}}_{\text{i}}+{\text{r}}_{\text{j}}\right)+\delta -\alpha_{\text{j}}\left({\text{c}}_{\text{i}}+{\text{c}}_{\text{j}}\right)<\text{0} \end{array}\right.\;$, $E_{\text{1}}(\text{0},\text{0})$ is the system evolutionary stable point, the local stability analysis at this time is shown in Table 3, and the phase diagram of the evolutionary path in Scene I is shown in Fig 2.

**Table 3:** Local stability analysis table for equilibrium point in scenario 1.

Balance point	Det(J)symbols	Tr(J)symbols	stability
$E_{\text{1}}(\text{0},\text{0})$	+	−	ESS
$E_{\text{2}}(\text{0},\text{1})$	−	inconclusive	saddle point (math.)
$E_{\text{3}}(\text{1},\text{0})$	−	inconclusive	saddle point (math.)
$E_{\text{4}}(\text{1},\text{1})$	+	+	point of instability (math.)

Evolutionary stabilization under such conditions results in the least favorable outcome for firms’ waste reduction cooperation, with neither firm willing to invest in R&D technology cooperation on waste reduction. This is because that when$\;\delta -\alpha_{\text{i}}\left({\text{c}}_{\text{i}}+{\text{c}}_{\text{j}}\right)<\text{0}\text{ or }\delta -\alpha_{\text{j}}\left({\text{c}}_{\text{i}}+{\text{c}}_{\text{j}}\right)<\text{0},$ the cost of waste reduction cooperation is too high for both firms and is higher than the penalty for non-cooperation δ. Moreover, $\beta_{\text{i}}\left({\text{r}}_{\text{i}}+{\text{r}}_{\text{j}}\right)+\delta -\alpha_{\text{i}}\left({\text{c}}_{\text{i}}+{\text{c}}_{\text{j}}\right)$ and${\;\beta }_{\text{j}}\left({\text{r}}_{\text{i}}+{\text{r}}_{\text{j}}\right)+\delta -\alpha_{\text{j}}\left({\text{c}}_{\text{i}}+{\text{c}}_{\text{j}}\right)$ are the revenue differences between the two enterprises’ cooperation and non-cooperation choice strategies under the cooperation strategy adopted by a certain enterprise, both of which are less than 0. Therefore, the enterprises will choose non-cooperation strategies based on revenue maximization, and the evolution of the system will eventually be stabilized in the state of (non-cooperation, non-cooperation), that is, (0,0).

**Fig 3:**
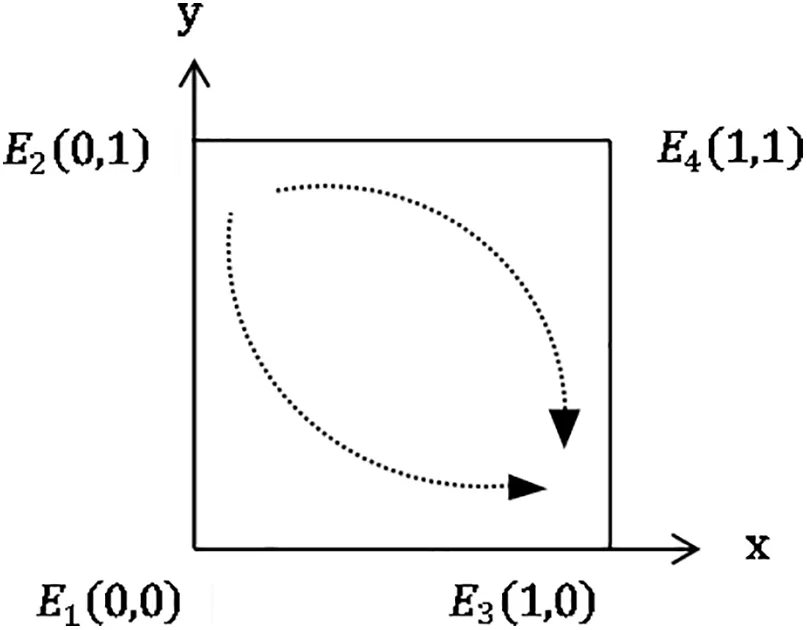
Evolutionary path phase diagram-Scenario 2.

(2) Scenario 2:

When$\left\{ \begin{array}{c} \delta -\alpha_{\text{i}}\left({\text{c}}_{\text{i}}+{\text{c}}_{\text{j}}\right)>\text{0}\\ \delta -\alpha_{\text{j}}\left({\text{c}}_{\text{i}}+{\text{c}}_{\text{j}}\right)<\text{0}\\ \beta_{\text{i}}\left({\text{r}}_{\text{i}}+{\text{r}}_{\text{j}}\right)+\delta -\alpha_{\text{i}}\left({\text{c}}_{\text{i}}+{\text{c}}_{\text{j}}\right)>\text{0}\\ \beta_{\text{j}}\left({\text{r}}_{\text{i}}+{\text{r}}_{\text{j}}\right)+\delta -\alpha_{\text{j}}\left({\text{c}}_{\text{i}}+{\text{c}}_{\text{j}}\right)<\text{0} \end{array}\right.\;$, ${\text{E}}_{\text{3}}(\text{1},\text{0})$ is the system evolutionary stability point, at this time, the local stability analysis is shown in Table 4, and the phase diagram of the evolutionary path at Scenario 2 is shown in Fig 3.

**Table 4:** Local stability analysis table for equilibrium point in scenario 2.

Balance point	Det(J)symbols	Tr(J)symbols	stability
$E_{\text{1}}(\text{0},\text{0})$	−	inconclusive	saddle point (math.)
$E_{\text{2}}(\text{0},\text{1})$	+	+	point of instability (math.)
$E_{\text{3}}(\text{1},\text{0})$	+	−	ESS
$E_{\text{4}}(\text{1},\text{1})$	−	inconclusive	saddle point (math.)

In this scenario, firm i is willing to cooperate in waste reduction, while firm j will adopt a non-cooperative strategy. For firm i, regardless of the strategy adopted by firm j, its benefits in both scenarios$\;\delta -\alpha_{\text{i}}\left({\text{c}}_{\text{i}}+{\text{c}}_{\text{j}}\right)$ and$\;\beta_{\text{i}}\left({\text{r}}_{\text{i}}+{\text{r}}_{\text{j}}\right)+\delta -\alpha_{\text{i}}\left({\text{c}}_{\text{i}}+{\text{c}}_{\text{j}}\right)$ are greater than 0. Moreover, the cost of waste reduction cooperation is$\;\alpha_{\text{i}}\left({\text{c}}_{\text{i}}+{\text{c}}_{\text{j}}\right)$ which is less than the penalty for default δ, so firm i will choose to adopt a kind of cooperative strategy. For enterprise j, when enterprise i adopts a cooperative strategy, the difference between the benefits of its cooperative and non-cooperative strategies is less than 0. The total benefit obtained by enterprise j from cooperative waste reduction is less than the waste reduction spillover benefits obtained from acting alone minus the penalty for breach of contract, so enterprise j will choose to exit from the cooperative network after obtaining the waste reduction spillover benefits from other enterprises and act alone, and the system’s evolution will ultimately stabilize in the (cooperative, non-cooperative) state, i.e., ${\text{E}}_{\text{3}}(\text{1},\text{0})$.

**Fig 4:**
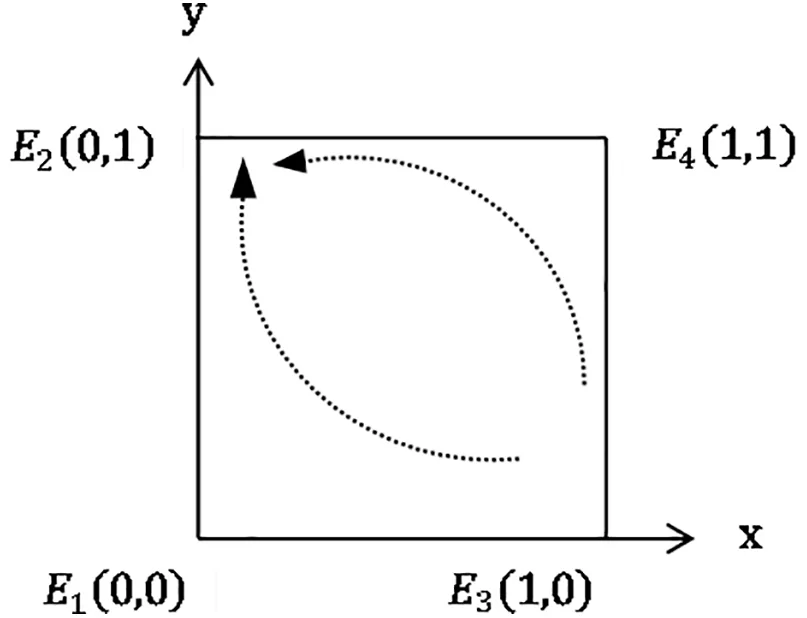
Evolutionary path phase diagram-Scenario 3.

(3) Scenario 3:

When$\left\{ \begin{array}{c} \delta -\alpha_{\text{i}}\left({\text{c}}_{\text{i}}+{\text{c}}_{\text{j}}\right)<\text{0}\\ \delta -\alpha_{\text{j}}\left({\text{c}}_{\text{i}}+{\text{c}}_{\text{j}}\right)>\text{0}\\ \beta_{\text{i}}\left({\text{r}}_{\text{i}}+{\text{r}}_{\text{j}}\right)+\delta -\alpha_{\text{i}}\left({\text{c}}_{\text{i}}+{\text{c}}_{\text{j}}\right)<\text{0}\\ \beta_{\text{j}}\left({\text{r}}_{\text{i}}+{\text{r}}_{\text{j}}\right)+\delta -\alpha_{\text{j}}\left({\text{c}}_{\text{i}}+{\text{c}}_{\text{j}}\right)>\text{0} \end{array}\right.\;$, ${\text{E}}_{\text{2}}(\text{0},\text{1})$ is the system evolutionary stability point, the local stability analysis at this time is shown in Table 5, and the phase diagram of the evolutionary path at scene 3 is shown in Fig 4.

**Table 5:** Local stability analysis of the equilibrium point in scene 3.

Balance point	Det(J)symbols	Tr(J)symbols	stability
$E_{\text{1}}(\text{0},\text{0})$	−	inconclusive	saddle point (math.)
$E_{\text{2}}(\text{0},\text{1})$	+	−	ESS
$E_{\text{3}}(\text{1},\text{0})$	+	+	point of instability (math.)
$E_{\text{4}}(\text{1},\text{1})$	−	inconclusive	saddle point (math.)

The evolutionary steady state of the system under Scenario 3 is that firm j is willing to cooperate with firm i in waste reduction, while firm i will choose a non-cooperative strategy. For firm j, regardless of the decision made by firm i, the difference between the benefits of firm j choosing the two strategies of cooperation and non-cooperation is greater than 0, and the cost ${\;\alpha }_{\text{j}}\left({\text{c}}_{\text{i}}+{\text{c}}_{\text{j}}\right)$ that firm j needs to pay to cooperate in waste reduction is smaller than the penalty δ for exiting the cooperative network. So firm j will choose to cooperate in waste reduction. For enterprise i, when enterprise j adopts the cooperative strategy, the difference between the revenue of enterprise i’s cooperative and non-cooperative strategies is less than 0. At this time, the total revenue obtained by enterprise i’s cooperative waste reduction is less than the difference which caused by the spillover benefits of waste reduction minus the penalty for breach of contract, when withdrawing from the cooperative network to act alone. Then the enterprise will choose to withdraw from the cooperative waste reduction network based on the maximization of revenue and obtain the spillover benefits of waste reduction from other enterprises. Eventually, the system will stabilize in the (non-cooperative, cooperative) state, i.e., ${\text{E}}_{\text{2}}(\text{0},\text{1})$ after a number of games.

**Fig 5:**
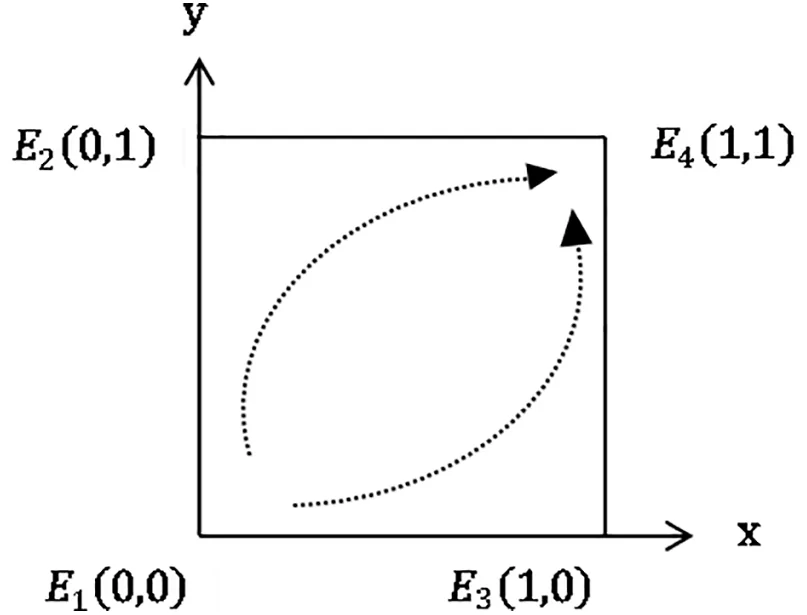
Evolutionary path phase diagram-Scenario 4.

(4) Scene 4:

When$\left\{ \begin{array}{c} \delta -\alpha_{\text{i}}\left({\text{c}}_{\text{i}}+{\text{c}}_{\text{j}}\right)>\text{0}\\ \delta -\alpha_{\text{j}}\left({\text{c}}_{\text{i}}+{\text{c}}_{\text{j}}\right)>\text{0}\\ \beta_{\text{i}}\left({\text{r}}_{\text{i}}+{\text{r}}_{\text{j}}\right)+\delta -\alpha_{\text{i}}\left({\text{c}}_{\text{i}}+{\text{c}}_{\text{j}}\right)>\text{0}\\ \beta_{\text{j}}\left({\text{r}}_{\text{i}}+{\text{r}}_{\text{j}}\right)+\delta -\alpha_{\text{j}}\left({\text{c}}_{\text{i}}+{\text{c}}_{\text{j}}\right)>\text{0} \end{array}\right.\;$,${\text{ E}}_{\text{4}}(\text{1},\text{1})$ is the system evolutionary stability point, at this time the local stability analysis is shown in Table 6, and the phase diagram of the evolutionary path at scene four is shown in Fig 5.

**Table 6:** Local stability analysis table of equilibrium point in scene 4.

Balance point	Det(J)symbols	Tr(J)symbols	stability
$E_{\text{1}}(\text{0},\text{0})$	+	+	Point of instability
$E_{\text{2}}(\text{0},\text{1})$	−	inconclusive	saddle point (math.)
$E_{\text{3}}(\text{1},\text{0})$	−	inconclusive	saddle point (math.)
$E_{\text{4}}(\text{1},\text{1})$	+	−	ESS

The evolutionary steady state of the system at Scenario 4 is optimal, when the difference between the benefits of enterprise i and enterprise j is greater than 0 regardless of whether they choose cooperative waste reduction or not. For enterprise i, the distributional benefit plus the waste reduction spillover benefit and minus the cooperation sharing cost gained from choosing cooperative waste reduction, which is greater than the total benefit from exiting the cooperative network; for enterprise j, the total benefit gained from choosing cooperative waste reduction is greater than the waste reduction spillover benefit minus the default penalty gained from exiting the cooperative network by acting alone, so the two enterprises choose cooperative waste reduction in order to gain more benefits, and the system evolution will eventually gradually stabilize at the ideal state of (cooperation, cooperation), i.e., ${\text{E}}_{\text{4}}(\text{1},\text{1})$.

**Fig 6:**
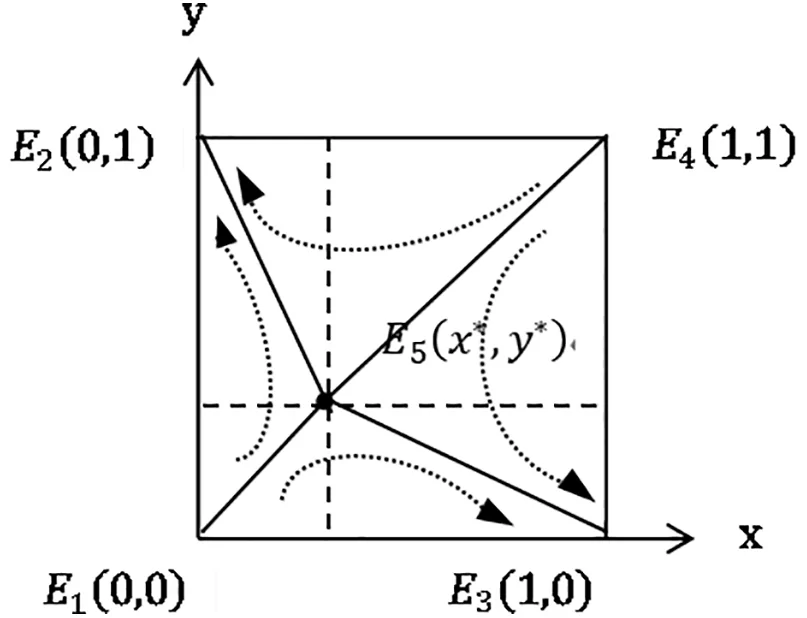
Evolutionary path phase diagram-Scenario 5.

(5) Scene 5:

$\text{when}\left\{ \begin{array}{c} \delta -\alpha_{\text{i}}\left({\text{c}}_{\text{i}}+{\text{c}}_{\text{j}}\right)>\text{0}\\ \delta -\alpha_{\text{j}}\left({\text{c}}_{\text{i}}+{\text{c}}_{\text{j}}\right)>\text{0}\\ \beta_{\text{i}}\left({\text{r}}_{\text{i}}+{\text{r}}_{\text{j}}\right)+\delta -\alpha_{\text{i}}\left({\text{c}}_{\text{i}}+{\text{c}}_{\text{j}}\right)<\text{0}\\ \beta_{\text{j}}\left({\text{r}}_{\text{i}}+{\text{r}}_{\text{j}}\right)+\delta -\alpha_{\text{j}}\left({\text{c}}_{\text{i}}+{\text{c}}_{\text{j}}\right)<\text{0} \end{array}\right.\;$, both equilibrium points ${\text{E}}_{\text{2}}(\text{0},\text{1}),{\text{E}}_{\text{3}}(\text{1},\text{0})\;$satisfy the stabilization conditions Det(J)>0 and Tr(J)<0, so${\text{ E}}_{\text{2}}(\text{0},\text{1})\text{ and }{\text{E}}_{\text{3}}(\text{1},\text{0})$ are both system evolutionary stable points, at this time, the local stability analysis is shown in Table 7, and the phase diagram of the evolutionary path at Scenario 5 is shown in Fig 6.

**Table 7:** Local stability analysis of equilibrium points in scene 5.

Balance point	Det(J)symbols	Tr(J)symbols	stability
$E_{\text{1}}(\text{0},\text{0})$	+	+	point of instability (math.)
$E_{\text{2}}(\text{0},\text{1})$	+	−	ESS
$E_{\text{3}}(\text{1},\text{0})$	+	−	ESS
$E_{\text{4}}(\text{1},\text{1})$	+	+	point of instability (math.)

The evolutionary stable state of the system at scenario 5 may be that enterprise i cooperates and enterprise j does not cooperate, or enterprise i does not cooperate and enterprise j cooperates. The specific evolutionary stable results are related to the initial choice probability values of the two enterprises. It can be seen from Fig 6.

When$\text{ x}>{\text{x}}^{*}$, $\text{y}<{\text{y}}^{*}$ (i.e., $\text{x}>\frac{\alpha_{\text{j}}\left({\text{c}}_{\text{i}}+{\text{c}}_{\text{j}}\right)-\delta }{\beta_{\text{j}}\left({\text{r}}_{\text{i}}+{\text{r}}_{\text{j}}\right)}$, $\text{y}<\frac{\alpha_{\text{i}}\left({\text{c}}_{\text{i}}+{\text{c}}_{\text{j}}\right)-\delta }{\beta_{\text{i}}\left({\text{r}}_{\text{i}}+{\text{r}}_{\text{j}}\right)}$), the probability that firm i chooses cooperative waste reduction is greater than the saddle point, while the probability that firm j chooses cooperative waste reduction is less than the saddle point, at this time, firm i is more inclined to choose cooperative waste reduction, and firm j is more inclined to choose to act alone, and eventually the evolution of the system will gradually stabilize at equilibrium point ${\text{E}}_{\text{3}}(\text{1},\text{0})$.

When $\text{x}<{\text{x}}^{*}$, $\text{y}>{\text{y}}^{*}$ (i.e., $\text{x}<\frac{\alpha_{\text{j}}\left({\text{c}}_{\text{i}}+{\text{c}}_{\text{j}}\right)-\delta }{\beta_{\text{j}}\left({\text{r}}_{\text{i}}+{\text{r}}_{\text{j}}\right)}$,$\text{ y}>\frac{\alpha_{\text{i}}\left({\text{c}}_{\text{i}}+{\text{c}}_{\text{j}}\right)-\delta }{\beta_{\text{i}}\left({\text{r}}_{\text{i}}+{\text{r}}_{\text{j}}\right)}$), the probability of enterprise j choosing cooperative waste reduction is greater than the saddle point, while the probability of enterprise i choosing cooperative waste reduction is less than the saddle point, at this time, enterprise j will choose cooperative waste reduction driven by the interests of enterprise j, and enterprise i is more inclined to choose to act alone, and eventually the evolution of the system will gradually stabilize at the equilibrium point ${\text{E}}_{\text{2}}(\text{0},\text{1})$.

When$\text{ x}<{\text{x}}^{*}$, $\text{ y}<{\text{y}}^{*}$ (i.e., $\text{x}<\frac{\alpha_{\text{j}}\left({\text{c}}_{\text{i}}+{\text{c}}_{\text{j}}\right)-\delta }{\beta_{\text{j}}\left({\text{r}}_{\text{i}}+{\text{r}}_{\text{j}}\right)}$, $\text{y}<\frac{\alpha_{\text{i}}\left({\text{c}}_{\text{i}}+{\text{c}}_{\text{j}}\right)-\delta }{\beta_{\text{i}}\left({\text{r}}_{\text{i}}+{\text{r}}_{\text{j}}\right)}$), the probability of both firms choosing to reduce waste through cooperation is less than the value at the saddle point, under these conditions both firms can not be sure what kind of choice to make, and after many games the system will eventually stabilize at equilibrium point ${\text{E}}_{\text{2}}(\text{0},\text{1})\text{ or}{\text{ E}}_{\text{3}}(\text{1},\text{0})$.

When $\text{x}>{\text{x}}^{*}$, $\text{y}>{\text{y}}^{*}$ (i.e., $\text{x}>\frac{\alpha_{\text{j}}\left({\text{c}}_{\text{i}}+{\text{c}}_{\text{j}}\right)-\delta }{\beta_{\text{j}}\left({\text{r}}_{\text{i}}+{\text{r}}_{\text{j}}\right)}$)$\text{ y}>\frac{\alpha_{\text{i}}\left({\text{c}}_{\text{i}}+{\text{c}}_{\text{j}}\right)-\delta }{\beta_{\text{i}}\left({\text{r}}_{\text{i}}+{\text{r}}_{\text{j}}\right)}$), the probability of both firms choosing to cooperate in waste reduction is greater than the value at the saddle point, under such conditions it is not possible to determine what choices the two firms will make, and the system will eventually stabilize at the equilibrium point after many games ${\text{E}}_{\text{2}}(\text{0},\text{1})\text{ or}{\text{ E}}_{\text{3}}(\text{1},\text{0}).$

**Fig 7:**
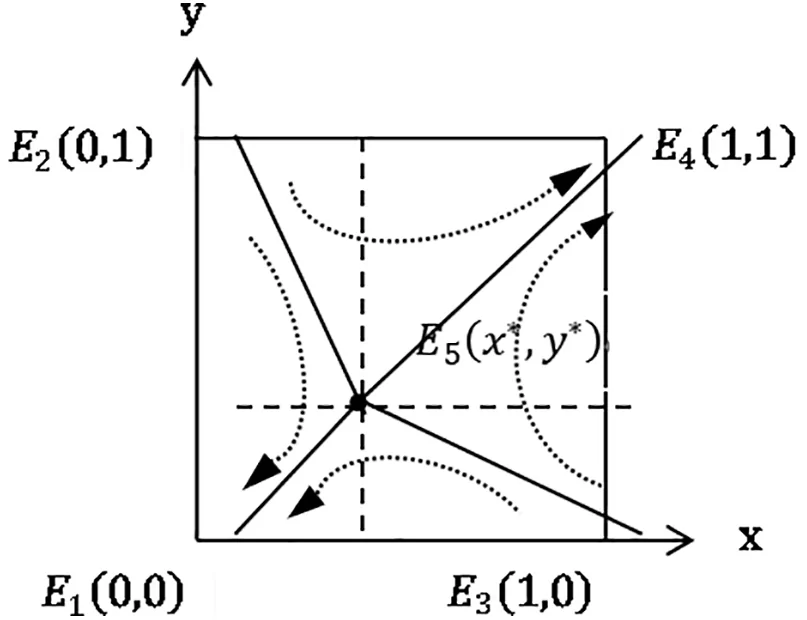
Evolutionary path phase diagram-Scenario 6.

(6) Scene 6:

When$\left\{ \begin{array}{c} \delta -\alpha_{\text{i}}\left({\text{c}}_{\text{i}}+{\text{c}}_{\text{j}}\right)<\text{0}\\ \delta -\alpha_{\text{j}}\left({\text{c}}_{\text{i}}+{\text{c}}_{\text{j}}\right)<\text{0}\\ \beta_{\text{i}}\left({\text{r}}_{\text{i}}+{\text{r}}_{\text{j}}\right)+\delta -\alpha_{\text{i}}\left({\text{c}}_{\text{i}}+{\text{c}}_{\text{j}}\right)>\text{0}\\ \beta_{\text{j}}\left({\text{r}}_{\text{i}}+{\text{r}}_{\text{j}}\right)+\delta -\alpha_{\text{j}}\left({\text{c}}_{\text{i}}+{\text{c}}_{\text{j}}\right)>\text{0} \end{array}\right.\;$,both equilibrium points ${\text{E}}_{\text{1}}(\text{0},\text{0}),{\text{E}}_{\text{4}}(\text{1},\text{1})$ satisfy the stabilization conditions of det(J)>0 and Tr(J)<0, so $\;{\text{E}}_{\text{1}}(\text{0},\text{0})\text{ and}{\text{ E}}_{\text{4}}(\text{1},\text{1})$ are both system evolutionary stable points, at this time, the local stability analysis is shown in Table 8, and the phase diagram of the evolutionary path at scene VI is shown in Fig 7.

**Table 8:** Local stability analysis of the equilibrium points in scene 6.

Balance point	Det(J)symbols	Tr(J)symbols	stability
$E_{\text{1}}(\text{0},\text{0})$	+	−	ESS
$E_{\text{2}}(\text{0},\text{1})$	+	+	point of instability (math.)
$E_{\text{3}}(\text{1},\text{0})$	+	+	point of instability (math.)
$E_{\text{4}}(\text{1},\text{1})$	+	−	ESS

The evolutionary stabilization outcome of the system at scenario 6 could be${\text{ E}}_{\text{1}}(\text{0},\text{0})$. That is to say, both firms are unwilling to choose to cooperate in waste reduction, and the evolutionary stabilization outcome may also be${\text{ E}}_{\text{4}}(\text{1},\text{1}).$ In other words, both enterprises are willing to cooperate, and the specific evolutionary stabilization result is related to the initial choice probability values of the two enterprises. As can be seen from Fig 7:

When$\text{ x}<{\text{x}}^{*}$, $\text{y}<{\text{y}}^{*}$ (i.e., $\text{x}<\frac{\alpha_{\text{j}}\left({\text{c}}_{\text{i}}+{\text{c}}_{\text{j}}\right)-\delta }{\beta_{\text{j}}\left({\text{r}}_{\text{i}}+{\text{r}}_{\text{j}}\right)}$,$\text{ y}<\frac{\alpha_{\text{i}}\left({\text{c}}_{\text{i}}+{\text{c}}_{\text{j}}\right)-\delta }{\beta_{\text{i}}\left({\text{r}}_{\text{i}}+{\text{r}}_{\text{j}}\right)}$), the probability of both firms choosing to cooperate in waste reduction is less than the value at the saddle point, both firms in this scenario are more willing to choose to give up their cooperation, and after continuous gaming, the system will eventually stabilize at the equilibrium point ${\text{E}}_{\text{1}}(\text{0},\text{0})$.

When$\text{ x}>{\text{x}}^{*}$, $\text{y}>{\text{y}}^{*}$ (i.e.,  .$\text{x}>\frac{\alpha_{\text{j}}\left({\text{c}}_{\text{i}}+{\text{c}}_{\text{j}}\right)-\delta }{\beta_{\text{j}}\left({\text{r}}_{\text{i}}+{\text{r}}_{\text{j}}\right)},\text{y}>\frac{\alpha_{\text{i}}\left({\text{c}}_{\text{i}}+{\text{c}}_{\text{j}}\right)-\delta }{\beta_{\text{i}}\left({\text{r}}_{\text{i}}+{\text{r}}_{\text{j}}\right)}$), the probability of both enterprises choosing cooperative waste reduction is greater than the value at saddle point, both firms are more willing to choose cooperative waste reduction in this scenario, and the system will eventually stabilize at the equilibrium point ${\text{E}}_{\text{4}}(\text{1},\text{1})$.

When$\text{ x}>{\text{x}}^{*}$, $\text{ y}<{\text{y}}^{*}$ (i.e., $\text{x}>\frac{\alpha_{\text{j}}\left({\text{c}}_{\text{i}}+{\text{c}}_{\text{j}}\right)-\delta }{\beta_{\text{j}}\left({\text{r}}_{\text{i}}+{\text{r}}_{\text{j}}\right)}$, $\text{y}<\frac{\alpha_{\text{i}}\left({\text{c}}_{\text{i}}+{\text{c}}_{\text{j}}\right)-\delta }{\beta_{\text{i}}\left({\text{r}}_{\text{i}}+{\text{r}}_{\text{j}}\right)}$), the probability of firm i choosing cooperative waste reduction is greater than the value at the saddle point, while the probability of firm j choosing cooperative waste reduction is less than the value at the saddle point. Under these conditions it is not possible to determine what choices both firms ultimately make, and ultimately the evolution of the system gradually stabilizes at equilibrium point ${\text{E}}_{\text{1}}(\text{0},\text{0})\text{ or }{\text{E}}_{\text{4}}(\text{1},\text{1})$.

When$\text{ x}<{\text{x}}^{*}$, $\text{y}>{\text{y}}^{*}$ (i.e., $\text{x}<\frac{\alpha_{\text{j}}\left({\text{c}}_{\text{i}}+{\text{c}}_{\text{j}}\right)-\delta }{\beta_{\text{j}}\left({\text{r}}_{\text{i}}+{\text{r}}_{\text{j}}\right)}$, $\text{y}>\frac{\alpha_{\text{i}}\left({\text{c}}_{\text{i}}+{\text{c}}_{\text{j}}\right)-\delta }{\beta_{\text{i}}\left({\text{r}}_{\text{i}}+{\text{r}}_{\text{j}}\right)}$), the probability of firm j choosing cooperative waste reduction is greater than the value at the saddle point, while the probability of firm i choosing cooperative waste reduction is less than the value at the saddle point, at this time, it is impossible to determine what kind of decision the two firms make, and eventually the evolution of the system will gradually stabilize at equilibrium point ${\text{E}}_{\text{1}}(\text{0},\text{0})\text{ or}{\text{ E}}_{\text{4}}(\text{1},\text{1})$.

#### 3.3.2 Discussion of factors influencing evolutionary outcomes.

Considering that the ultimate goal of this article is to make the enterprises of all parties have a greater willingness to cooperate in waste reduction by adopting various reasonable measures, and eventually to make the outcome of the game lastingly stable in the ideal state of (cooperation, cooperation), and that any of the two parties to the game choosing not to cooperate means that the cooperation in waste reduction is facing a failure, so for the six scenarios mentioned above, the in-depth discussion focuses on two possibilities for the last scenario. In order to facilitate the later calculation and analysis, assume that the total cost$\text{ C}={\text{c}}_{\text{i}}+{\text{c}}_{\text{j}}$, total benefit$\text{ R}={\text{r}}_{\text{i}}+{\text{r}}_{\text{j}}$, where each parameter should satisfy the scenario 6 $\left\{ \begin{array}{c} \delta -\alpha_{\text{i}}\left({\text{c}}_{\text{i}}+{\text{c}}_{\text{j}}\right)<\text{0}\\ \delta -\alpha_{\text{j}}\left({\text{c}}_{\text{i}}+{\text{c}}_{\text{j}}\right)<\text{0}\\ \beta_{\text{i}}\left({\text{r}}_{\text{i}}+{\text{r}}_{\text{j}}\right)+\delta -\alpha_{\text{i}}\left({\text{c}}_{\text{i}}+{\text{c}}_{\text{j}}\right)>\text{0}\\ \beta_{\text{j}}\left({\text{r}}_{\text{i}}+{\text{r}}_{\text{j}}\right)+\delta -\alpha_{\text{j}}\left({\text{c}}_{\text{i}}+{\text{c}}_{\text{j}}\right)>\text{0} \end{array}\right.\;$ conditions.

From the phase diagram of the evolution path in Fig 7, it can be seen that there are two evolutionary outcomes for both firms, i.e., eventually both firms choose to cooperate in waste reduction and the system gradually stabilizes at point${\text{ E}}_{\text{4}}(\text{1},\text{1})$ or both enterprises adopt the non-cooperative strategy and act alone, and the system gradually stabilizes at point ${\text{E}}_{\text{1}}(\text{0},\text{0})$. The folding line ${\text{E}}_{\text{2}}\;{\text{E}}_{\text{5}}\;{\text{E}}_{\text{3}}$ is the dividing line between the quadrilateral ${\text{E}}_{\text{2}}{\text{E}}_{\text{1}}{\text{E}}_{\text{3}}{\text{E}}_{\text{5}}$ and the quadrilateral ${\text{E}}_{\text{2}}{\text{E}}_{\text{5}}{\text{E}}_{\text{3}}{\text{E}}_{\text{4}}$ whose area determines the final evolutionary direction of the system. Let the area of the quadrilateral ${\text{E}}_{\text{2}}{\text{E}}_{\text{1}}{\text{E}}_{\text{3}}{\text{E}}_{\text{5}}$ is ${\text{S}}_{\text{1}}$ and the area of the quadrilateral${\text{ E}}_{\text{2}}{\text{E}}_{\text{5}}{\text{E}}_{\text{3}}{\text{E}}_{\text{4}}\;$ is${\text{ S}}_{\text{2}}$, when${\text{ S}}_{\text{1}}>{\text{S}}_{\text{2}}$, the probability that both firms choose not to cooperate is greater than the probability of cooperation; when${\text{ S}}_{\text{1}}<{\text{S}}_{\text{2}}$, the probability that both firms choose to cooperate is greater than the probability that they do not cooperate; when$\;{\text{ S}}_{\text{1}}={\text{S}}_{\text{2}}$,the probability that both enterprises choose cooperation or not is equal. If we want to analyze the impact of various influence factors upon the strategy choices of both enterprises, the impact analysis of each factor upon the ${\text{S}}_{\text{2}}$ size can be simplified. From Fig 7, it can be seen that ${\text{S}}_{\text{2}}$ which stands for the area of ${\text{E}}_{\text{2}}{\text{E}}_{\text{5}}{\text{E}}_{\text{3}}{\text{E}}_{\text{4}}$ can be obtained as follows.


$$\begin{array}{c} S_{\text{2}}=\text{1}-S_{\text{1}}=\text{1}-\frac{\text{1}}{\text{2}}x^{*}-\frac{\text{1}}{\text{2}}y^{*}=\text{1}-\frac{\text{1}}{\text{2}}\left[\frac{\alpha_{j}\left({\text{c}}_{\text{i}}+{\text{c}}_{\text{j}}\right)-\delta }{\beta_{j}\left(r_{i}+r_{j}\right)}+\frac{\alpha_{i}\left({\text{c}}_{\text{i}}+{\text{c}}_{\text{j}}\right)-\delta }{\beta_{i}\left(r_{i}+r_{j}\right)}\right] \end{array}$$ (10)

(1) Impact of total cost on evolutionary outcomes

By using the area formula of$\;{\text{S}}_{\text{2}}$, the first-order partial derivatives which stands for ${\text{ S}}_{\text{2}}$ with respect to the total cost C can be shown below.


$$\begin{array}{c} \frac{\partial S_{\text{2}}}{\partial \text{C}}=-\frac{\text{1}}{\text{2}}\left[\frac{\alpha_{j}}{\beta_{j}\left(r_{i}+r_{j}\right)}+\frac{\alpha_{i}}{\beta_{i}\left(r_{i}+r_{j}\right)}\right]<\text{0} \end{array}$$ (11)

Eq. (11) indicates that as the increasement of cooperation total cost between the parties, the area ${\text{S}}_{\text{2}}$of region${\text{ E}}_{\text{2}}{\text{E}}_{\text{5}}{\text{E}}_{\text{3}}{\text{E}}_{\text{4}}$ gradually becomes smaller, the probability that both enterprises choosing waste reduction cooperation also becomes smaller, and the total cost has a negative correlation with the area of region ${\text{E}}_{\text{2}}{\text{E}}_{\text{5}}{\text{E}}_{\text{3}}{\text{E}}_{\text{4}}$. With the increasement of input cost, the willingness of choosing cooperation decreases as for the two sides, that is to say, by controlling the input cost, there is a rising inclination in the probability of reducing waste cooperation. After many games, the system is gradually stabilized at the$\;{\text{E}}_{\text{4}}(\text{1},\text{1})$.

(2) Impact of total returns on evolutionary outcomes

By using the area formula of$\;{\text{S}}_{\text{2}}$, the first-order partial derivatives which stands for ${\text{ S}}_{\text{2}}$ with respect to the total return R can be shown below.


$$\begin{array}{c} \frac{\partial S_{\text{2}}}{\partial \text{R}}=\frac{\text{1}}{\text{2}}\left\{\frac{\beta_{j}\left[\alpha_{i}\left({\text{c}}_{\text{i}}+{\text{c}}_{\text{j}}\right)-\delta \right]}{\beta_{j}^{\text{2}}{\text{R}}^{\text{2}}}+\frac{\beta_{i}\left[\alpha_{j}\left({\text{c}}_{\text{i}}+{\text{c}}_{\text{j}}\right)-\delta \right]}{\beta_{i}^{\text{2}}{\text{R}}^{\text{2}}}\right\}>\text{0} \end{array}$$ (12)

From the positive and negative first order partial derivatives above, it can be seen that as the total benefit of the cooperation between the two parties increases, the area${\text{ S}}_{\text{2}}$ of the region ${\text{E}}_{\text{2}}{\text{E}}_{\text{5}}{\text{E}}_{\text{3}}{\text{E}}_{\text{4}}$ gradually becomes larger, and the probability of both enterprises choosing waste reduction cooperation also gradually becomes larger, and the total benefit has a positive correlation with the area of the region${\text{E}}_{\text{2}}{\text{E}}_{\text{5}}{\text{E}}_{\text{3}}{\text{E}}_{\text{4}}$. The total benefit is positively related to the area of the region. The more the total benefit of the cooperation between the two enterprises to reduce waste, the more enterprises tend to choose the cooperation to reduce waste, so we can promote the cooperation between the two sides by increasing the benefit of the cooperation to reduce waste, and the system will be stabilized in the ideal state of$E_{\text{4}}(\text{1},\text{1})$ The system will be gradually stabilized in the ideal state.

(3) Impact of default penalty coefficients on evolutionary outcomes

By the formula of the area $S_{\text{2}}$, the first order partial derivative representing $S_{\text{2}}$ to the default penalty coefficient δ is shown below:


$$\begin{array}{c} \frac{\partial S_{\text{2}}}{\partial \delta }=\frac{\text{1}}{\text{2}}\left(\frac{\text{1}}{\beta_{j}\left(r_{i}+r_{j}\right)}+\frac{\text{1}}{\beta_{i}\left(r_{i}+r_{j}\right)}\right)>\text{0} \end{array}$$ (13)

From the above derivation formula, the first-order partial derivative representing the area${{\;S}_{\text{2}}\text{ of region E}}_{\text{2}}{\text{E}}_{\text{5}}{\text{E}}_{\text{3}}{\text{E}}_{\text{4}}\;$ to the default penalty coefficient δ is greater than 0, indicating that as the default penalty coefficient increases, the area${\text{ S}}_{\text{2}}$ of region$\;{\text{E}}_{\text{2}}{\text{E}}_{\text{5}}{\text{E}}_{\text{3}}{\text{E}}_{\text{4}\;}$ is larger, the probability that both enterprises choose to cooperate in waste reduction is increasing, and the default penalty coefficient is positively correlated with the area of the region ${\text{E}}_{\text{2}}{\text{E}}_{\text{5}}{\text{E}}_{\text{3}}{\text{E}}_{\text{4}}$. The larger the default penalty coefficient is, the default cost for enterprises to give up cooperation will increase, and then enterprises are more inclined to cooperate in waste reduction, so the cooperation can be promoted between the two sides by appropriately increasing the default penalty, and finally make the system evolution gradually stabilize at${\;E}_{\text{4}}(\text{1},\text{1})$.

(4) Impact of cost-sharing coefficients on evolutionary outcomes

Since the sum of the cost-sharing coefficients of the two parties to the game is 1, i.e.,$\alpha_{i}+\alpha_{j}=\text{1}$, so for the sake of convenience, let $\alpha_{i}=\alpha$, then${\;\alpha }_{j}=\text{1}-\alpha$. According to the area formula of $S_{\text{2}}$, the partial derivative of${\;S}_{\text{2}}$ with respect to the cost-sharing coefficients is shown below:


$$\begin{array}{c} \frac{\partial S_{\text{2}}}{\partial \alpha }=\frac{\text{1}}{\text{2}}\left[\frac{\alpha \left({\text{c}}_{\text{i}}+{\text{c}}_{\text{j}}\right)}{\beta_{i}\left(r_{i}+r_{j}\right)}-\frac{(\text{1}-\alpha )\left({\text{c}}_{\text{i}}+{\text{c}}_{\text{j}}\right)}{\beta_{j}\left(r_{i}+r_{j}\right)}\right] \end{array}$$ (14)

$$\begin{array}{c} \frac{\partial^{\text{2}}S_{\text{2}}}{\partial \alpha^{\text{2}}}=\frac{\text{1}}{\text{2}}\left[\frac{\left(c_{i}+c_{j}\right)}{\beta_{i}\left(r_{i}+r_{j}\right)}+\frac{\left(c_{i}+c_{j}\right)}{\beta_{j}\left(r_{i}+r_{j}\right)}\right]>\text{0} \end{array}$$ (15)

The first-order partial derivative function of the cost-sharing coefficient from$\;S_{\text{2}}$,it can be seen that the effect resulting from cost-sharing coefficient α to the area$S_{\text{2}}$of region ${\text{E}}_{\text{2}}{\text{E}}_{\text{5}}{\text{E}}_{\text{3}}{\text{E}}_{\text{4}}$ in the scenario of controlling for other variables is not monotonous, and the relationship cannot be directly determined by the first-order partial derivatives, so the second-order partial derivatives are further sought. From the second-order partial derivative formula, it is known that the second-order partial derivative from the area${\;S}_{\text{2}}$ to the cost-sharing coefficient α is greater than 0, so${\;S}_{\text{2}}$ is a concave function with respect to the cost-sharing coefficient, so that $S_{\text{2}}$ exists a minimal value of$\;S_{min}$. Let$\;\frac{\partial S_{\text{2}}}{\partial \alpha }=\text{0}$, the equation value${\;\alpha }^{*}$ can be solved and then the minimal value of$\;S_{\text{2}}$ will be found. At this time, the probability that two sides choose to reduce-waste cooperation is smallest, the system will eventually converge to the${\;E}_{\text{1}}(\text{0},\text{0})$, so the cost-sharing coefficient based on $\alpha^{*}$ is suggested to adjusted, so as to increase the probability of cooperation between the two sides of the game.

(5) Impact of benefit distribution coefficients on evolutionary outcomes

Since the coefficients sum of the benefit distribution between the game’s two sides to 1, i.e.,$\beta_{i}+\beta_{j}=\text{1}$, in order to facilitate the calculation, let$\;\beta_{i}=\beta$, then$\;\beta_{j}=\text{1}-\beta$. By the area formula of ${\;S}_{\text{2}}$, the partial derivative of${\;S}_{\text{2}}$ with respect to the coefficient of benefit distribution is shown as below:


$$\begin{array}{c} \frac{\partial S_{\text{2}}}{\partial \beta }=-\frac{\text{1}}{\text{2}}\left\{\frac{\left[\alpha_{i}\left({\text{c}}_{\text{i}}+{\text{c}}_{\text{j}}\right)-\delta \right]}{(\text{1}-\beta {)}^{\text{2}}\left(r_{i}+r_{j}\right)}-\frac{\left[\alpha_{j}\left({\text{c}}_{\text{i}}+{\text{c}}_{\text{j}}\right)-\delta \right]}{\beta^{\text{2}}\left(r_{i}+r_{j}\right)}\right\} \end{array}$$ (16)

$$\begin{array}{c} \frac{\partial^{\text{2}}S_{\text{2}}}{\partial \beta^{\text{2}}}=-\left[\frac{\alpha_{i}\left(c_{i}+c_{j}\right)-\delta }{(\text{1}-\beta {)}^{\text{3}}\left(r_{i}+r_{j}\right)}+\frac{\alpha_{j}\left(c_{i}+c_{j}\right)-\delta }{\beta^{\text{3}}\left(r_{i}+r_{j}\right)}\right]<\text{0} \end{array}$$ (17)

Obviously, the first order partial derivative function from $S_{\text{2}}$ to the benefit distribution coefficients shows that the benefit distribution coefficient β for the area${\;S}_{\text{2}}$ of region ${\text{E}}_{\text{2}}{\text{E}}_{\text{5}}{\text{E}}_{\text{3}}{\text{E}}_{\text{4}}$ is not monotonous in the scenario controlling for other variables and the relationship cannot be directly determined by the first-order partial derivatives, so the second-order partial derivatives are further sought. From the second-order partial derivative formula, it is known that the second order partial derivative of $S_{\text{2}}$ to the benefit distribution coefficient β is less than 0. As a convex function, there exists an extremely large value of${\;S}_{max}$. Let$\;\frac{\partial S_{\text{2}}}{\;\partial \beta }=\text{0}$, the equation value$\;\beta^{*}\;$ and the maximum value can be solved. At this time, the cooperation probability of waste reducing for the two sides is the largest, after continuous game the system will eventually be toward the equilibrium point$\;E_{\text{4}}(\text{1},\text{1})$. The benefit distribution coefficient β can be adjusted as close as possible to$\;\beta^{*}$ in order to increase the cooperation willingness of both sides.

The above analyzes the effect of each parameter in the revenue matrix on the $S_{\text{2}}$, where the total cost C is negatively correlated with${\;S}_{\text{2}}$, the total return R and the default penalty coefficient δ are positively related with $S_{\text{2}}$, the cost sharing coefficient α and the benefit sharing coefficient β show a non-monotonic relationship with $S_{\text{2}}$. However, during the stability analysis of the evolutionary game, the influence mechanism resulting from the waste reduction spillover effect coefficient μ on the$\;S_{\text{2}}$ is not very clear, and it is necessary to carry through further analysis through the evolution simulation of complex network node behavior.

## 4 Analysis and discussion of network evolution simulation results

### Ethics Statement

This study is a theoretical research based on evolutionary game model and numerical simulation, which does not involve human participants, animal experiments, field investigation or clinical research. Therefore, ethical approval and informed consent were not required for this study.

### 4.1 Network simulation steps

In the first step, when t=0,first a scale-free network that conforms to the characteristics of waste reduction cooperation needs to be constructed and then initially the parameters of the network may be set, in which the strategies chosen by the network nodes are automatically assigned by the system according to the set of game strategies.

In the second step, when t=1,each node in the network plays the first game with $P_{i}(t)$ probability of playing the first game with neighboring nodes and randomly selects the strategy to be adopted based on the set of game strategies.

In the third step, when t=2, the network node randomly compares its gains with those of its neighboring nodes, the node does not make a strategy change when its own gain is more than that of its neighboring nodes, and when its own gain is less than that of its neighboring nodes, the node mimics the strategy of its neighboring nodes with probability W.

In the fourth step, when t=3, after mimicked the strategy of neighboring nodes, it continues to compare the gain with neighboring nodes. If the gain is still low, the node will continue the game, and the nodes in the network that have not reached a stable state will disconnect the connecting edges with the original neighboring nodes and reestablish the connecting edges with other nodes with $V_{ij}$ probability.

In the fifth step, the above steps are repeated until all the nodes in the network reach stability and end the simulation operation.

### 4.2 Initial parameterization

Here, the urban waste reduction network cooperation model can be constructed, in which the horizontal axis represents the number of network evolution game, and the vertical axis represents the evolution depth, the influence mechanism of influencing factor parameters such as cost sharing coefficients on network evolution may be studied and discussed. In the game experiment, the number of tests t is set to 2000 in advance, and then the simulation test is carried out based on the different sizes of cooperative networks such as 50, 100, and 500 node sizes.The horizontal axis represents the number of evolutionary game rounds, and the vertical axis represents the cooperation probability of the network nodes. To improve the readability of the figures, only the first 300 rounds are displayed in the evolutionary trajectory figures because the major changes in cooperation probability mainly occur during the early stage of evolution. The convergence state and stability of each trajectory were evaluated using the complete 2000-round simulation results, and the detailed assessment results are provided in Appendix A, Table A1. In the simulation experiment, the speed of the network curve changes to quantify the evolution of the cooperative network, the stability point of the cooperative network evolution game is represented by the numbers 0 and 1. When the evolution of the cooperative network is stable at 0, it represents the stable state of the evolution for the enterprise to choose the “non-cooperation” strategy, and when the evolution result is stable at 1, it represents the stable state of the evolution for the enterprise to choose the “cooperation” strategy. When the evolution result is finally stabilized at 1, it represents the evolutionarily stable state for enterprises to choose the “cooperation” strategy. Referring to the existing research and the literature on cooperative networks in other fields [33,34], the initial values of the parameters in the cooperative model of enterprise waste reduction network are set. The parameters of subject i are set as follows. Make the waste reduction cost $c_{i}$ invested by subject *i* equal to 4, make the waste reduction benefit $r_{i}$ of subject *i* equal to 10, make $\gamma_{i}$ equal to 10 which stands for the benefit of subject i’s own waste reduction resources, make benefit sharing coefficient $\beta_{i}$ equal to 0.5, make cost-sharing coefficient $\alpha_{i}$ equal to 0.5, make spillover effect coefficient $\mu_{i}$ equal to 0.15, make default penalty coefficient δ equal to 0.6.

### 4.3 Impact of cost-sharing coefficients on the evolution of cooperative networks

It is easy to know that the cost of waste reduction refers to all the costs incurred in the process of producing a certain product, from the initial procurement of raw materials to the final recycling and disposal [35], to reducing the generation of solid waste, prevent and solve the pollution of solid waste to the environment, and promote the intelligent construction of waste management system. Then, enterprises in the initial stage of waste reduction cooperation to reduce the cost of waste reduction input, waste reduction costs whether to input, the amount of input will affect the behavior of cooperation between enterprises to reduce waste, and thus the size of the cost-sharing coefficient will have a direct impact on the interests of the enterprises and cooperative relations, but also on the results of the evolution of the entire cooperation network.

**Fig 8:**
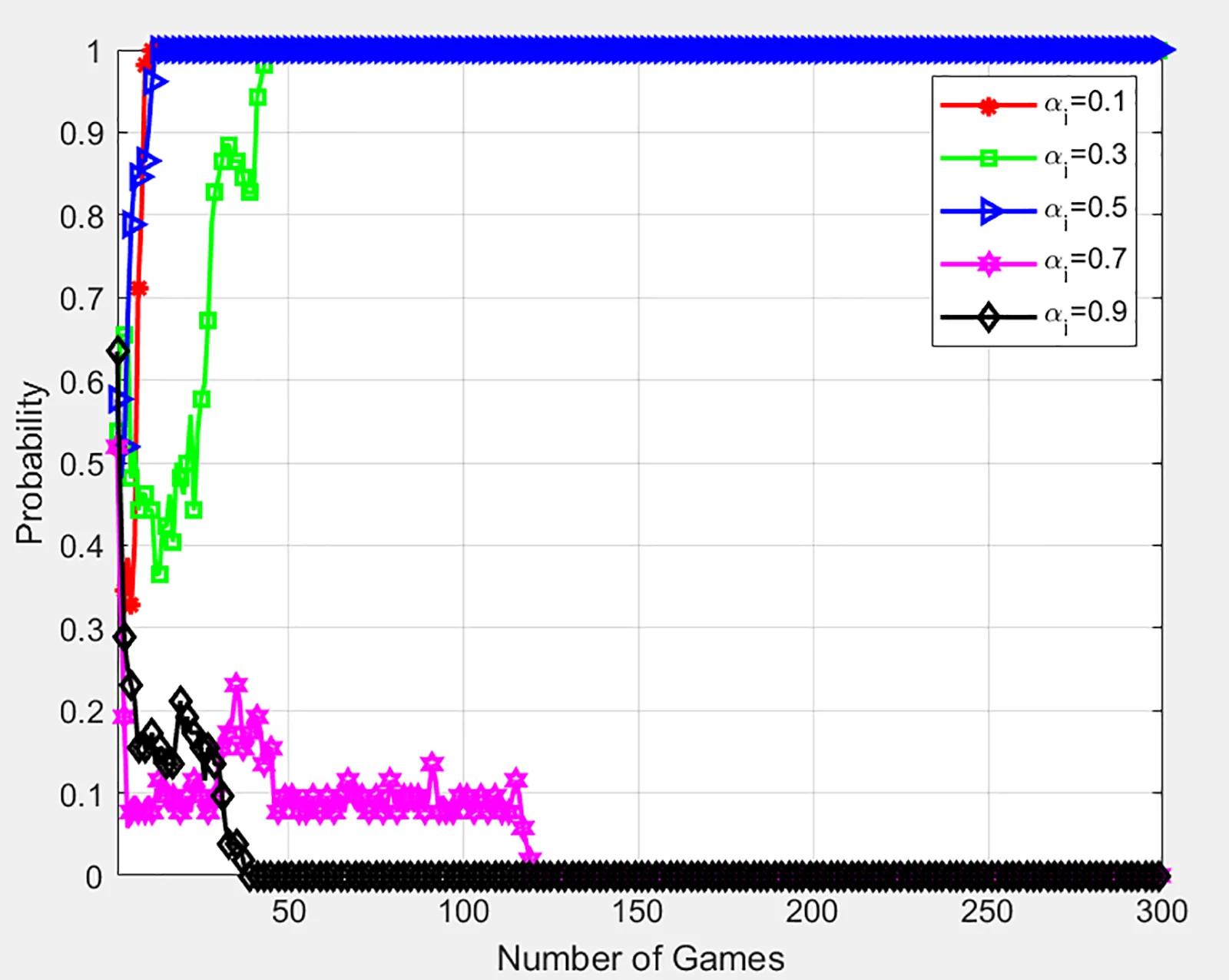
Effect of cost sharing factor on network evolution at 50 nodes.

**Fig 9:**
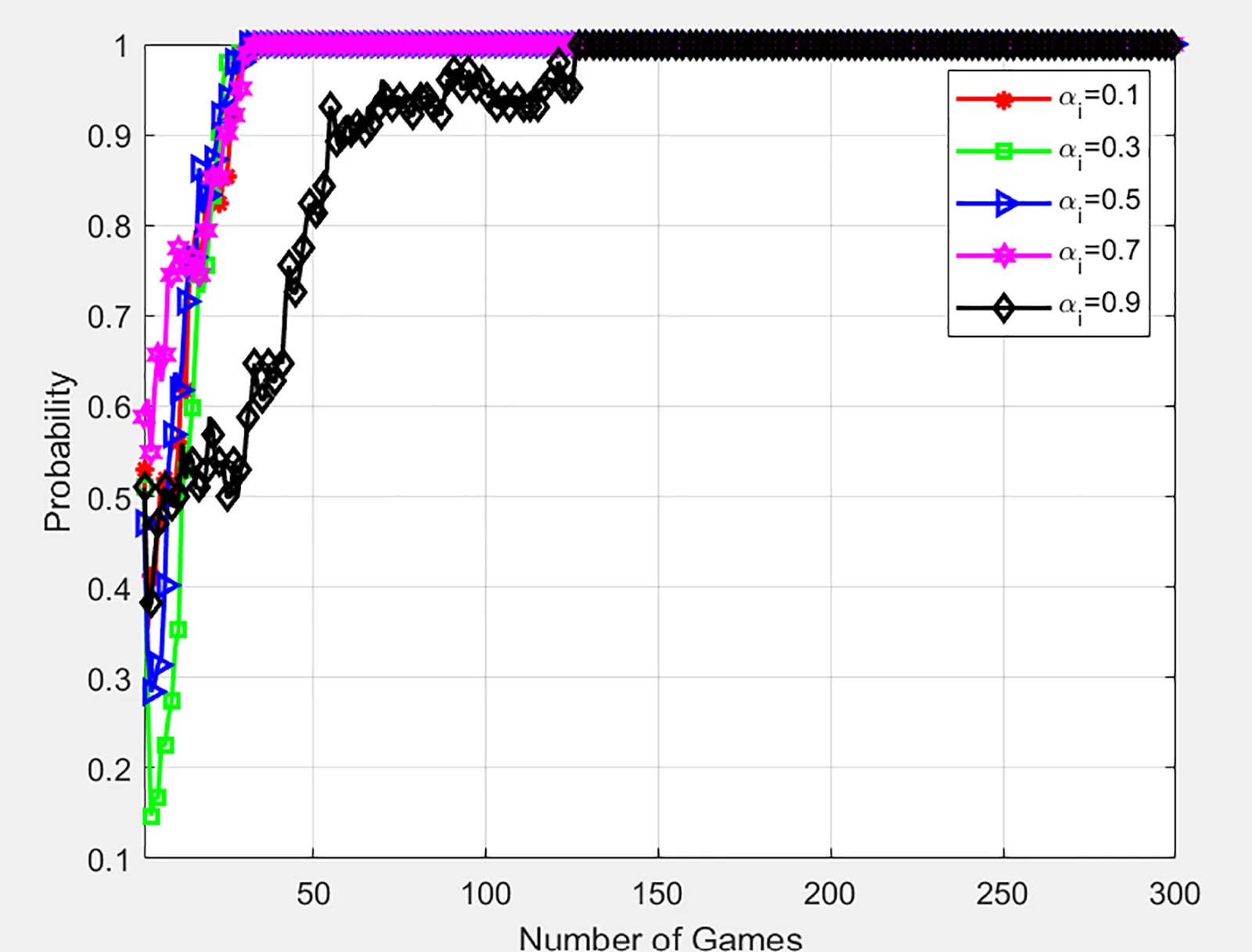
Effect of cost sharing factor on network evolution at 100 nodes.

**Fig 10:**
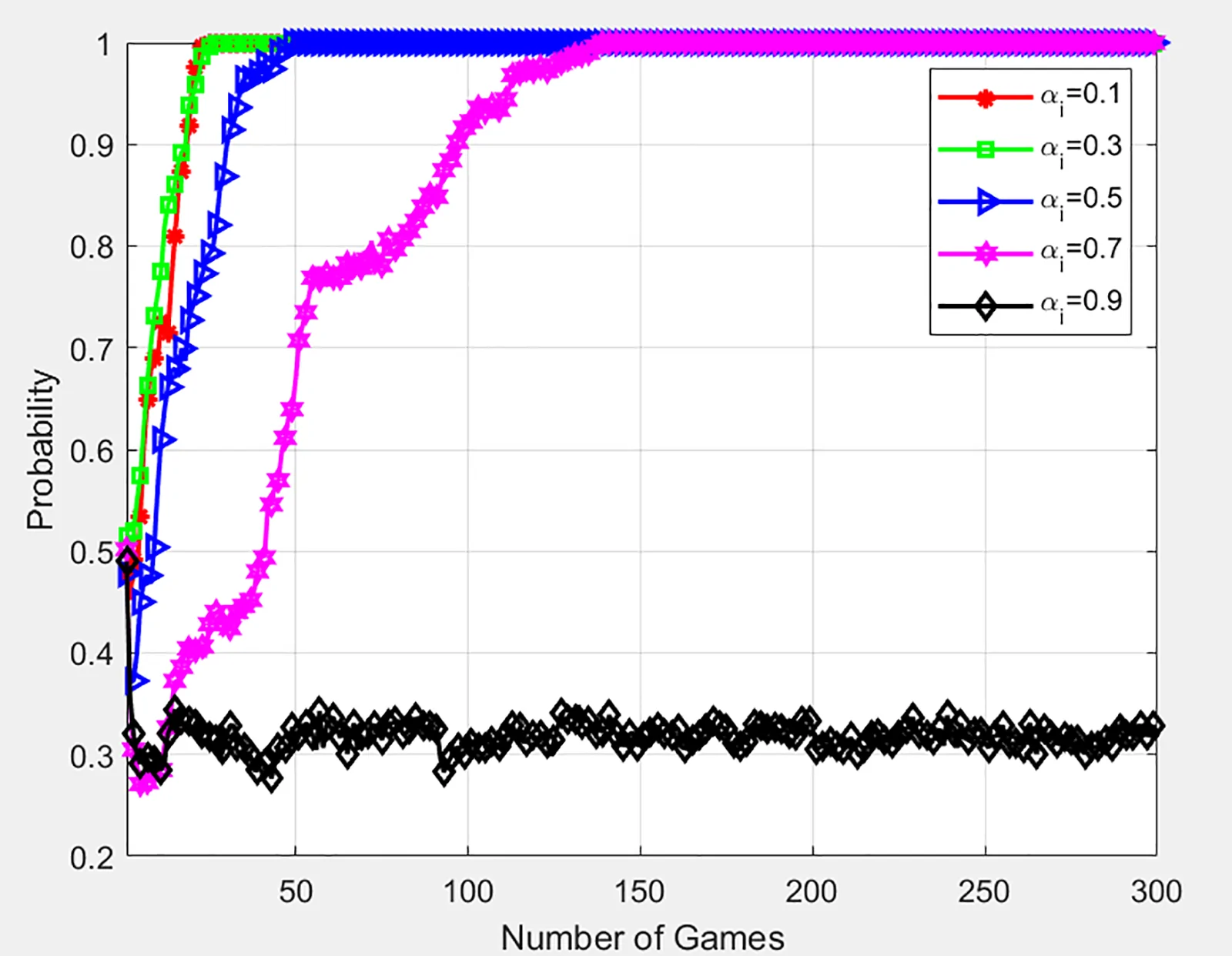
Effect of cost sharing factor on network evolution at 500 nodes.

To more accurately analyze the impact of the cost-sharing coefficient on the cooperative behavior of enterprises in waste reduction, the parameters other than the fixed cost-sharing coefficient are kept unchanged, and drawing on Yin’s practice in the study of environmental cost management of the supply chain of the paper industry [36], the changes in the value of the cost-sharing coefficient are set here as shown in the following Table 9, and the results of the evolution of the cooperation in the waste-reduction network obtained by simulation are shown in Figs 8–10.

**Table 9:** Parameterization of cost-sharing coefficients for cooperative behavior in municipal waste reduction.

network size	Cost-sharing factor	network size	Cost-sharing factor	network size	Cost-sharing factor
50	0.1	100	0.1	500	0.1
50	0.3	100	0.3	500	0.3
50	0.5	100	0.5	500	0.5
50	0.7	100	0.7	500	0.7
50	0.9	100	0.9	500	0.9

Note for Figs 8–10: The simulations were run for 2000 rounds; only the first 300 rounds are shown for clarity. The convergence state was assessed based on the complete 2000-round simulation results.

By observing the evolutionary simulation results in Figs 8–10, it can be seen that when other parameters remain unchanged, changing the cost-sharing coefficient will affect both the evolution direction and the evolution speed of the enterprise waste reduction cooperation network. Overall, when the cost-sharing coefficient is relatively low or moderate, enterprises bear a smaller proportion of waste reduction costs, and the cooperation probability of the network is more likely to evolve toward 1. When the cost-sharing coefficient is relatively high, the cost pressure borne by enterprises increases, which weakens their willingness to participate in waste reduction cooperation and slows down the diffusion of cooperative strategies.

In the small-scale waste reduction cooperation network with 50 nodes, when the cost-sharing coefficient is 0.1, 0.3, and 0.5, the cooperation probability eventually stabilizes at 1, indicating that enterprises are more inclined to choose the “cooperation” strategy under a relatively reasonable cost-sharing level. However, when the cost-sharing coefficient increases to 0.7 and 0.9, the cooperation probability eventually stabilizes at 0. This shows that in a small-scale cooperative network, excessive cost-sharing will significantly increase the burden of enterprises. Since the number of participating enterprises is relatively small, the choice of non-cooperation can spread more quickly among neighboring enterprises. As a result, enterprises tend to give up cooperation to protect their own interests, and the network finally evolves toward a non-cooperative stable state.

In the 100-node cooperative network, the cooperation probability under different cost-sharing coefficients eventually tends to stabilize at 1. However, when the cost-sharing coefficient is relatively high, especially when it reaches 0.9, the evolution curve fluctuates for a longer period and the convergence speed becomes slower. In the 500-node cooperative network, the evolution speed is slower than that of the 50-node and 100-node networks. When the cost-sharing coefficient is 0.7, the cooperation probability still evolves toward 1, but the convergence process is obviously delayed. When the cost-sharing coefficient is 0.9, the cooperation probability remains at a relatively low level for a long time in the displayed evolutionary process, indicating that an excessively high cost-sharing coefficient is not conducive to the formation of stable waste reduction cooperation in large-scale networks.

In summary, when the network scale is small, an excessive cost-sharing coefficient may directly lead the cooperation network to evolve toward 0. In medium- and large-scale waste reduction cooperation networks, a higher cost-sharing coefficient will slow down the evolution speed of the cooperation network, increase the fluctuation of the evolutionary process, and may even make the cooperation probability remain at a low level for a long time. Therefore, the cost-sharing coefficient should be controlled within a reasonable range. Only by reducing the excessive cost burden of enterprises can the willingness of enterprises to participate in waste reduction cooperation be improved and the stable evolution of the waste reduction cooperation network be promoted.

### 4.4 Impact of default penalty coefficients on the evolution of cooperative networks

**Fig 11:**
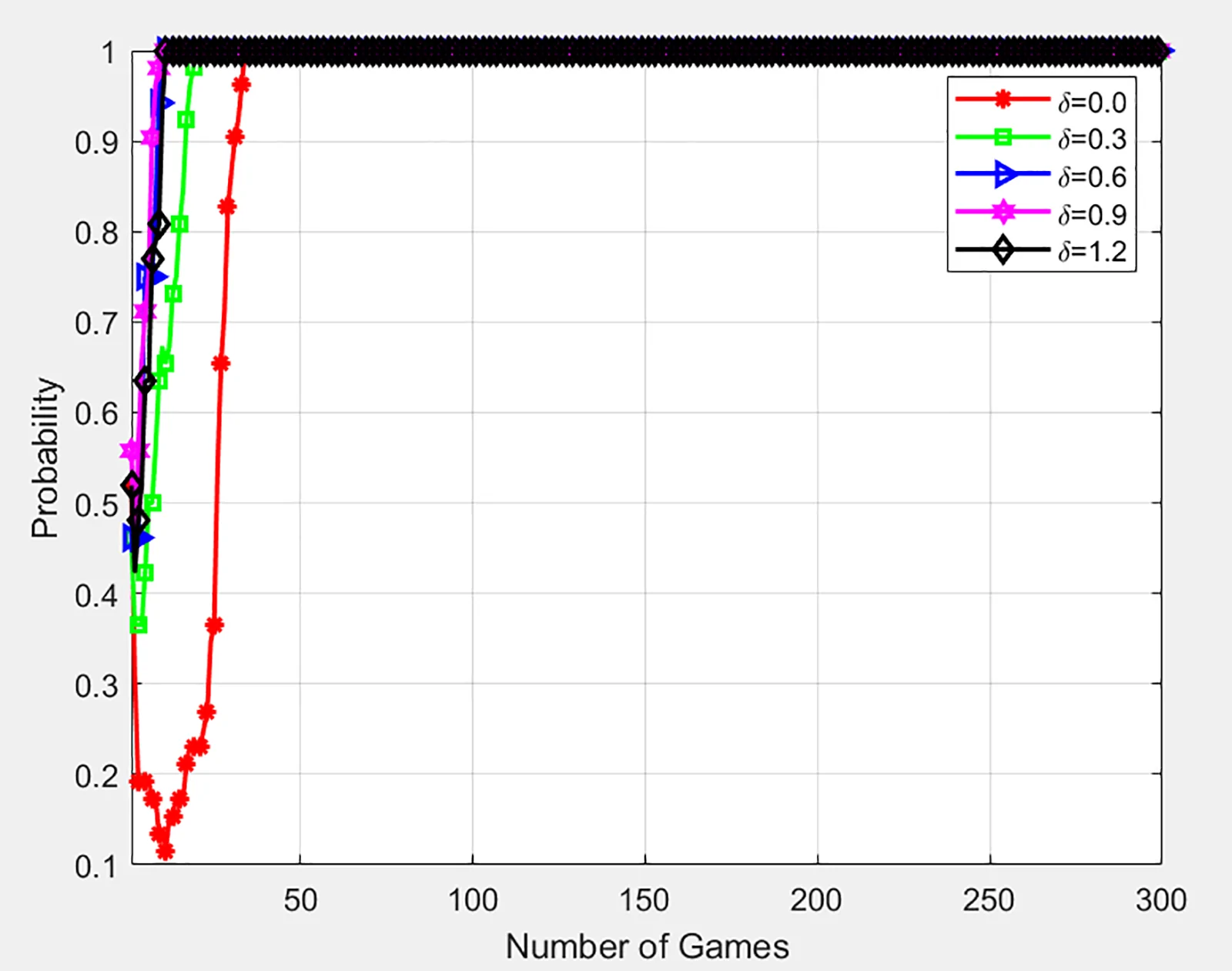
Effect of default penalty coefficient on network evolution at 50 nodes.

**Fig 12:**
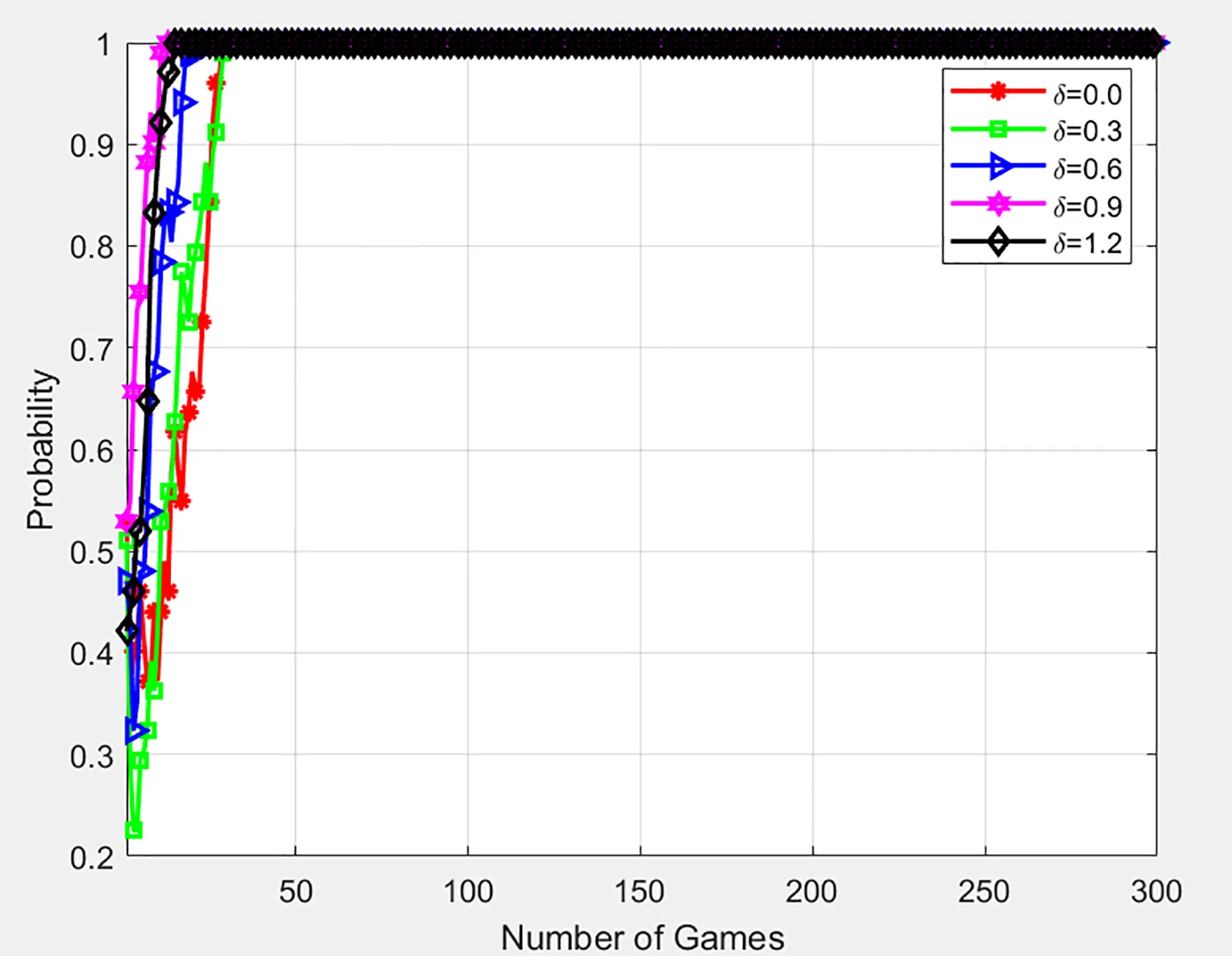
Effect of default penalty coefficient on network evolution at 100 nodes.

**Fig 13:**
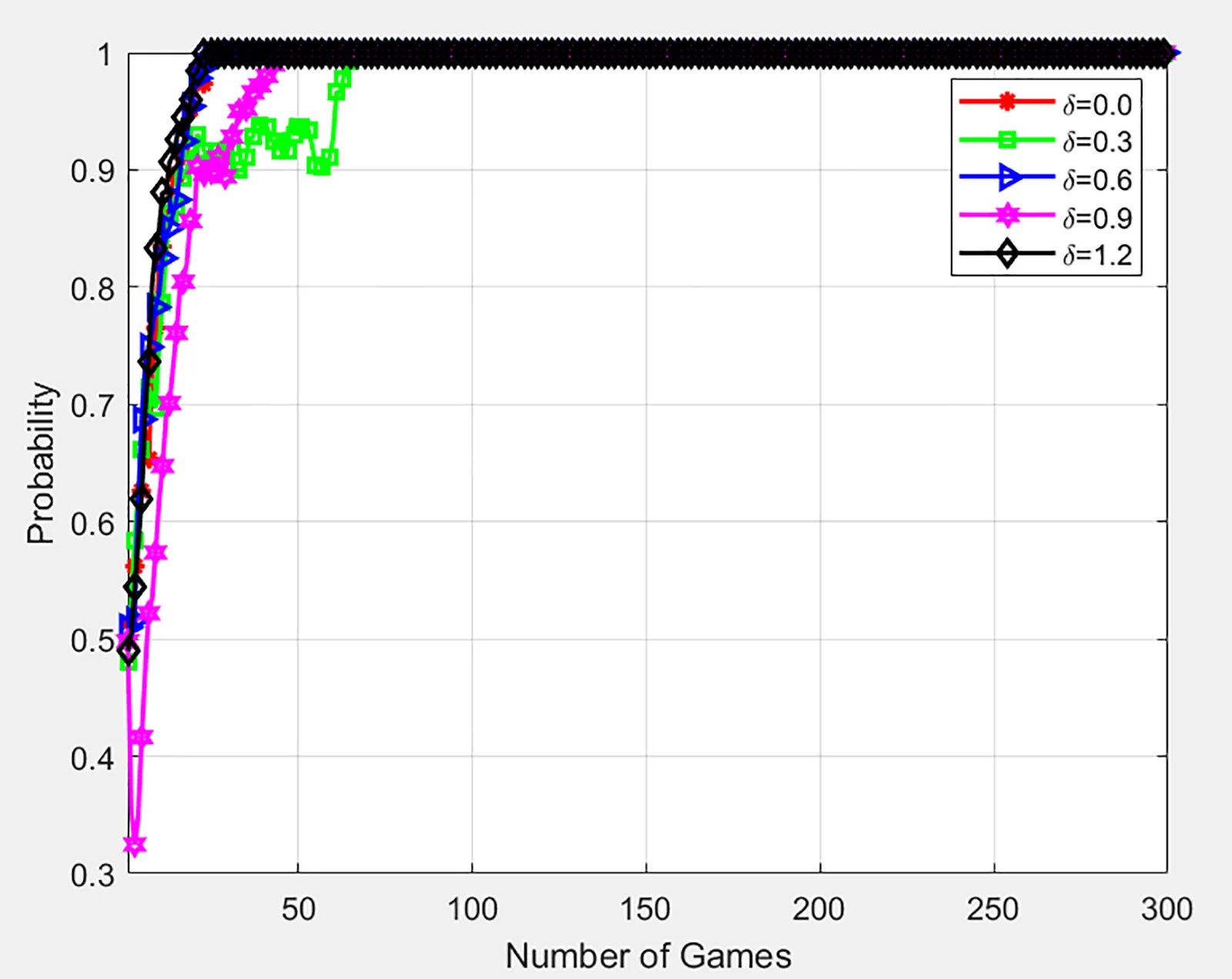
Effect of default penalty coefficient on network evolution at 500 nodes.

In the process of waste reduction cooperation, enterprises reach a certain cooperation agreement through mutual negotiation [37], and if one party violates the agreement, it will be punished, and the violating party will be punished to make up for the losses of the cooperative party. The penalty mechanism is mainly used to monitor the waste reduction cooperation of enterprises, through the establishment of a reasonable penalty system, it can reduce the default behavior of enterprises participating in the waste reduction cooperation, and encourage enterprises to fulfill their obligations to reduce waste, to promote the waste reduction cooperation [38]. Penalty mechanisms will have an impact on the waste reduction cooperation behavior between enterprises, in the absence of certain punishment constraints, it is easy for some enterprises to produce the idea of free-riding, really pay a lot of investment, on the one hand, did not get in line with the expected benefits, on the other hand, paid a certain amount of cost of waste reduction, and in the long run, it will reduce the enthusiasm of the enterprises to participate in the cooperation of waste reduction and affect the efficiency of the enterprise’s cooperation of waste reduction. To analyze the impact of the default penalty coefficient on the cooperative behavior of enterprises in waste reduction, drawing on the practice of Shi in the study of the impact of default penalties on the low-carbon cooperative behavior of the construction supply chain [39], the parameters other than the fixed default penalty coefficient remain unchanged, and the change in the value of the default penalty coefficient is shown in Table 10. In the cooperative network of different sizes, the study coefficient size of the impact of the evolution of the cooperation of enterprises in the waste reduction network, and the results of the specific evolutionary simulation are shown in Figs 11–13.

**Table 10:** Parameterization of penalty coefficients for non-compliance with municipal waste reduction cooperation acts.

network size	Penalty coefficient for non-compliance	network size	Penalty coefficient for non-compliance	network size	Penalty coefficient for non-compliance
50	0	100	0	500	0
50	0.3	100	0.3	500	0.3
50	0.6	100	0.6	500	0.6
50	0.9	100	0.9	500	0.9
50	1.2	100	1.2	500	1.2

Note for Figs 11–13: The simulations were run for 2000 rounds; only the first 300 rounds are shown for clarity. The convergence state was assessed based on the complete 2000-round simulation results.

From the evolutionary simulation results in Figs 11–13, it can be seen that in the three scales of waste reduction cooperation networks, when the default penalty coefficient is 0, the cooperation network can still evolve toward the stable cooperative state, but the convergence speed is relatively slower and the evolution curve fluctuates more obviously in the early stage. When the default penalty coefficient is greater than 0, the evolution curve generally reaches the stable cooperative state faster, indicating that the existence of the default penalty coefficient can promote inter-enterprise waste reduction cooperation. In the cooperation networks with 50 nodes and 100 nodes, the number of enterprise subjects involved in waste reduction cooperation is relatively small, so the strategy information selected by each subject at a certain moment spreads faster among enterprises. With the introduction and increase of the default penalty coefficient, the network evolution curve tends to stabilize faster. When individual enterprises choose the “non-cooperation” strategy, the penalty mechanism increases the cost of default and weakens the attractiveness of opportunistic behavior, thereby increasing the probability of enterprises choosing waste reduction cooperation.

In the waste reduction cooperation network with 500 nodes, the evolution curves under different default penalty coefficients eventually evolve and stabilize at 1. Compared with the 50-node and 100-node networks, the change of network scale has less influence on the final evolution result, but it affects the speed of network evolution and the fluctuation of the early evolutionary process. The larger the network scale, the more enterprises participate in the cooperation network, and the slower the propagation speed of information and strategy learning in the whole cooperation network. Therefore, some evolution curves in the 500-node network need a relatively longer time to reach the stable cooperative state. After the default penalty coefficient is introduced, enterprises face higher costs when choosing the “non-cooperation” strategy, which can effectively restrain default behavior and promote the diffusion of cooperative strategies.

In summary, in the process of enterprise waste reduction cooperation, it is necessary to establish a certain penalty mechanism to constrain enterprises’ waste reduction cooperation behavior, so that more enterprises can choose the “cooperation” strategy and promote inter-enterprise waste reduction cooperation. At the same time, the default penalty coefficient should be controlled within a reasonable range, especially in large-scale networks. A reasonable penalty mechanism can reduce free-riding and default behavior, improve enterprises’ willingness to participate in waste reduction cooperation, and promote the stable evolution of the waste reduction cooperation network.

### 4.5 Impact of benefits distribution coefficients on the evolution of cooperative networks

**Fig 14:**
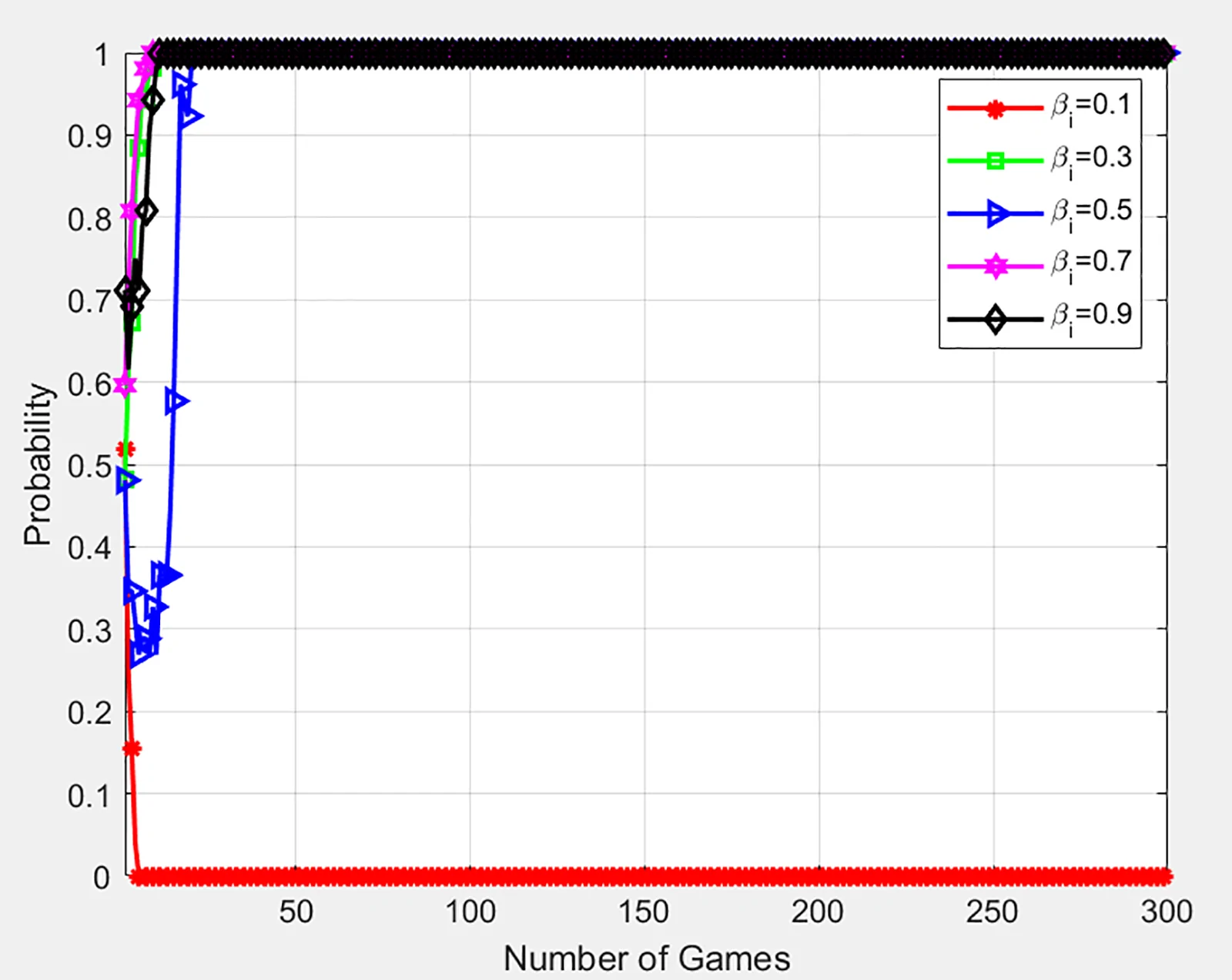
Effect of benefit distribution coefficients on network evolution at 50 nodes.

**Fig 15:**
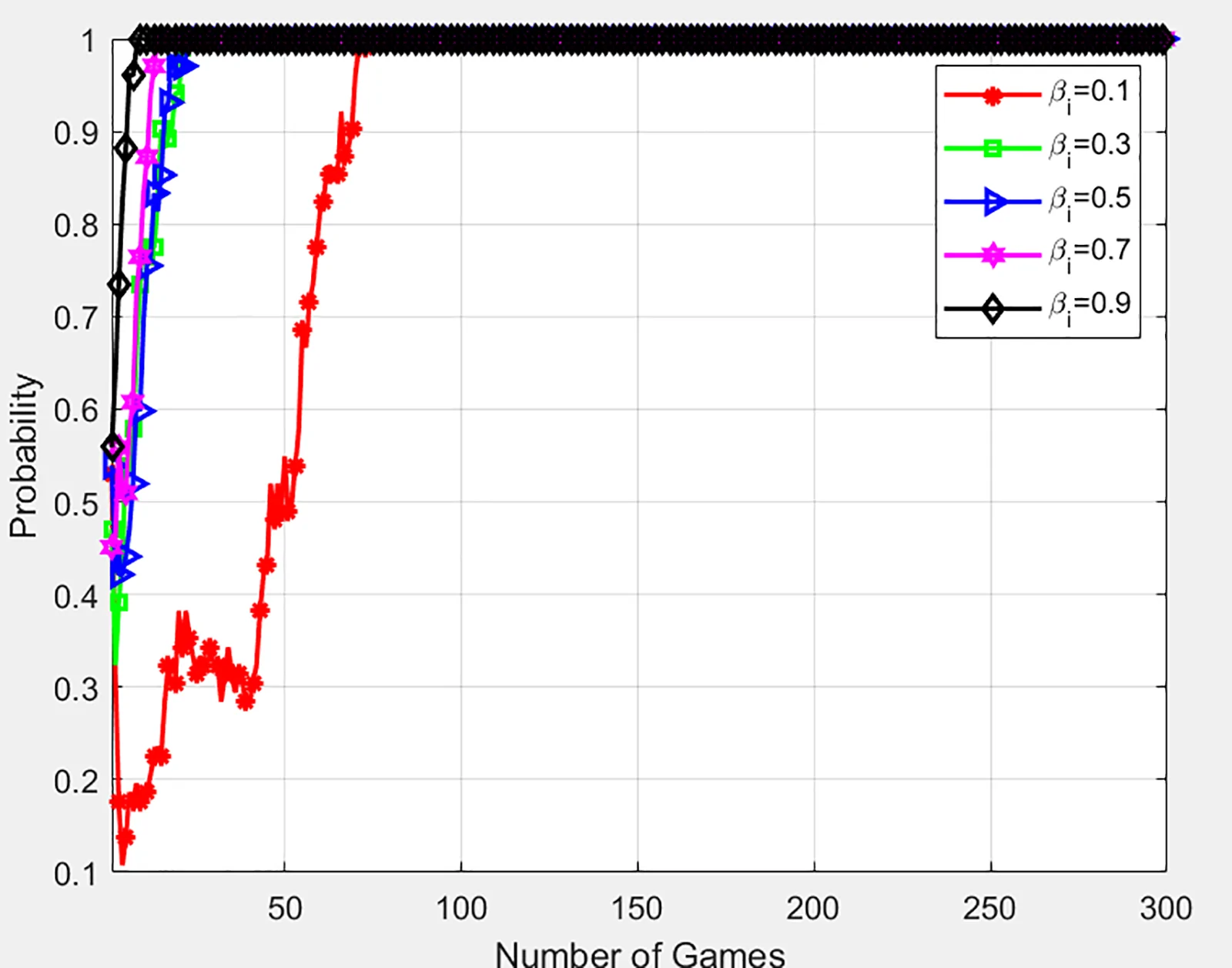
Effect of benefit distribution coefficient on network evolution at 100 nodes.

**Fig 16:**
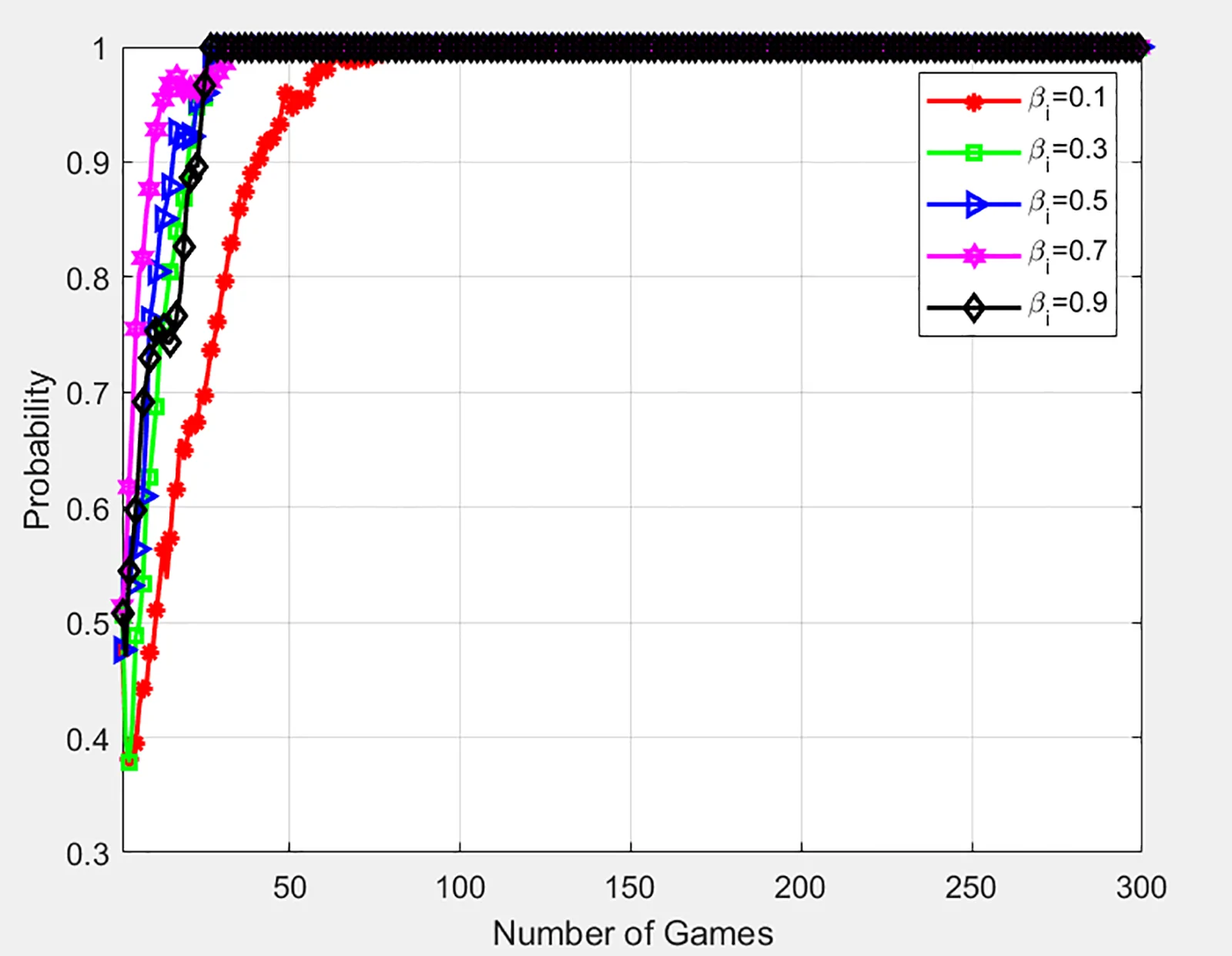
Effect of benefit distribution coefficient on network evolution at 500 nodes.

The choice of waste reduction cooperation by participating enterprises is complex, because there are big differences in culture and system between different enterprises, and it is difficult to analyze the implementation of waste reduction by enterprises with specific data, and the reasonableness of the distribution of the benefits obtained from the cooperation will affect the development of the cooperation between enterprises in the field of waste reduction [40]. The distribution of benefits plays a key role in the behavior of waste reduction cooperation, if enterprises do not get the expected benefits, it will reduce the willingness of enterprises to continue to cooperate in waste reduction so that the efficiency of cooperation between enterprises will be reduced, which will lead to the failure of waste reduction cooperation [41]. Reasonable benefit distribution is also a basic condition to ensure long-term cooperation among nodes, so benefit distribution is an important influencing factor of waste reduction cooperation behavior. To study the influence of the size of the benefit allocation coefficient on the evolution results of the cooperation network, the parameters other than the benefit allocation coefficient are fixed unchanged, and we draw on the practice of Zhou et al. in the study of the influence of the benefit allocation factors on the cooperative network of collaborative innovation of products [26], and we set the change of the value of the benefit allocation coefficient to be shown in Table 11, and we discuss the evolution of the three scales of cooperation network respectively, and the specific evolution simulation results are shown in Figs 14–16.

**Table 11:** Parameterization of benefit distribution coefficients for cooperative behavior in municipal waste reduction.

network size	Benefit-sharing coefficients	network size	Benefit-sharing coefficients	network size	Benefit-sharing coefficients
50	0.1	100	0.1	500	0.1
50	0.3	100	0.3	500	0.3
50	0.5	100	0.5	500	0.5
50	0.7	100	0.7	500	0.7
50	0.9	100	0.9	500	0.9

Note for Figs 14–16: The simulations were run for 2000 rounds; only the first 300 rounds are shown for clarity. The convergence state was assessed based on the complete 2000-round simulation results.

By observing the evolution simulation results in Figs 14–16, it can be seen that the benefit distribution coefficient has an important impact on the evolution of the waste reduction cooperation network. In the 50-node waste reduction cooperation network, when the benefit distribution coefficient is 0.1, the evolution result finally converges to 0, indicating that an excessively small benefit distribution coefficient will reduce enterprises’ willingness to participate in waste reduction cooperation and lead enterprises to choose the strategy of “non-cooperation”. When the benefit distribution coefficient is 0.3, 0.5, 0.7, and 0.9, the cooperation probability eventually evolves and stabilizes at 1, which indicates that a relatively reasonable benefit distribution coefficient is conducive to promoting cooperative behavior among enterprises.

In the 100-node and 500-node cooperative networks, the evolution results under different benefit distribution coefficients finally tend to stabilize at 1. However, when the benefit distribution coefficient is relatively small, especially when it is 0.1, the evolution curve fluctuates more obviously in the early stage and the convergence speed is slower. This shows that although the increase of network scale can enhance the diffusion capacity of cooperative strategies and reduce the possibility of the network finally evolving toward non-cooperation, an excessively small benefit distribution coefficient will still slow down the evolution of the cooperation network and increase the difficulty of cooperation formation. Compared with the 50-node network, the 100-node and 500-node networks contain more participating enterprises and more connection paths, so the cooperative strategy is more likely to spread in the network and eventually promote the cooperation probability to stabilize at 1.

In general, a fair and reasonable distribution of benefits is more conducive to the evolution of the cooperation network. The size of the benefit distribution coefficient should be kept within a reasonable range to promote enterprise waste reduction cooperation. If the benefit distribution coefficient is too small, enterprises may not obtain sufficient benefits from cooperation, which will weaken their willingness to participate in waste reduction cooperation and may even make the evolution result tend to 0 in small-scale networks. Therefore, corporations should formulate a reasonable benefit distribution plan that meets the interests of all parties, so as to improve enterprises’ willingness to cooperate and ensure the long-term stability of waste reduction cooperation.

### 4.6 Impact of waste reduction spillovers on the evolution of cooperative networks

**Fig 17:**
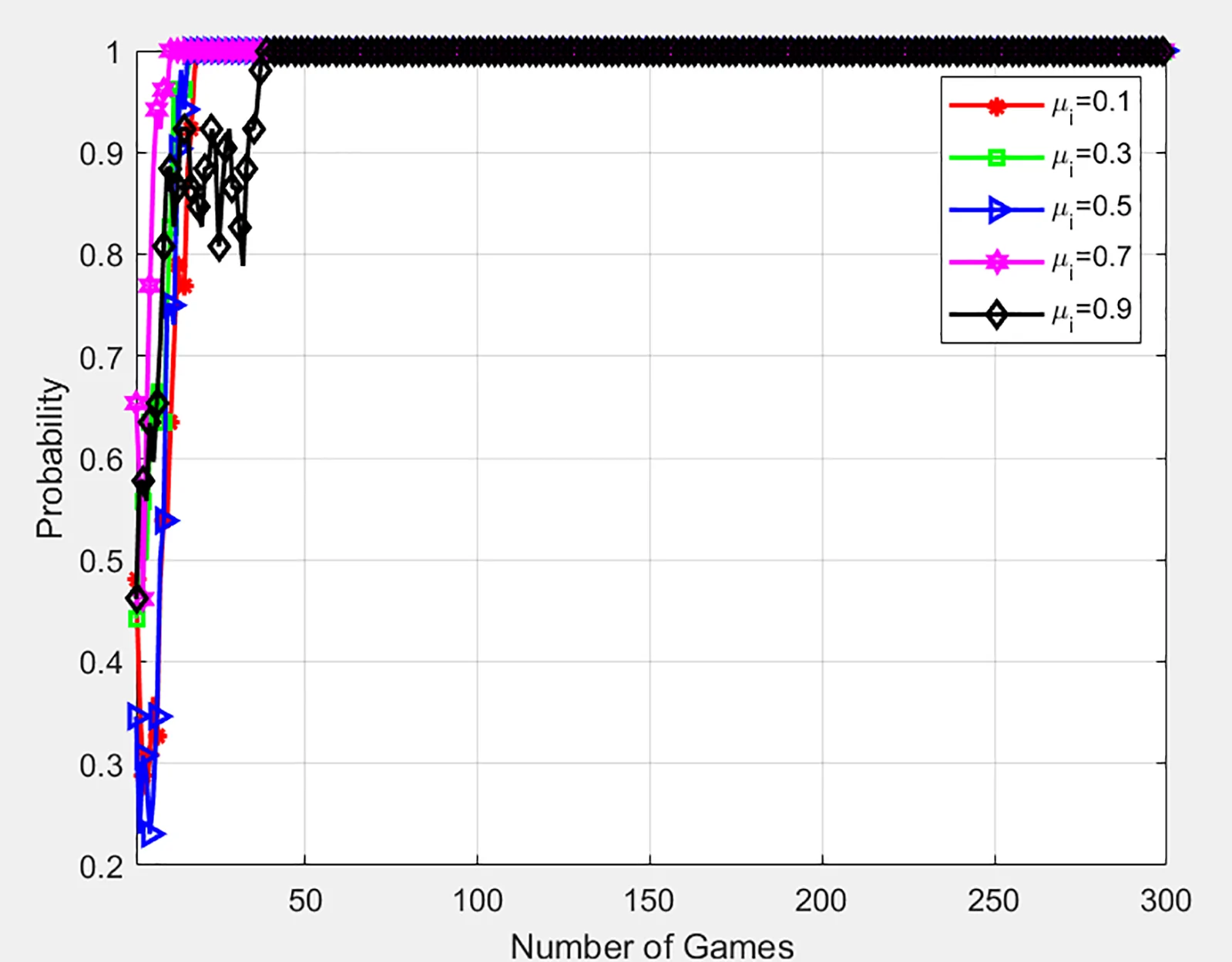
Impact of waste reduction spillover effect coefficient on network evolution at 50 nodes.

**Fig 18:**
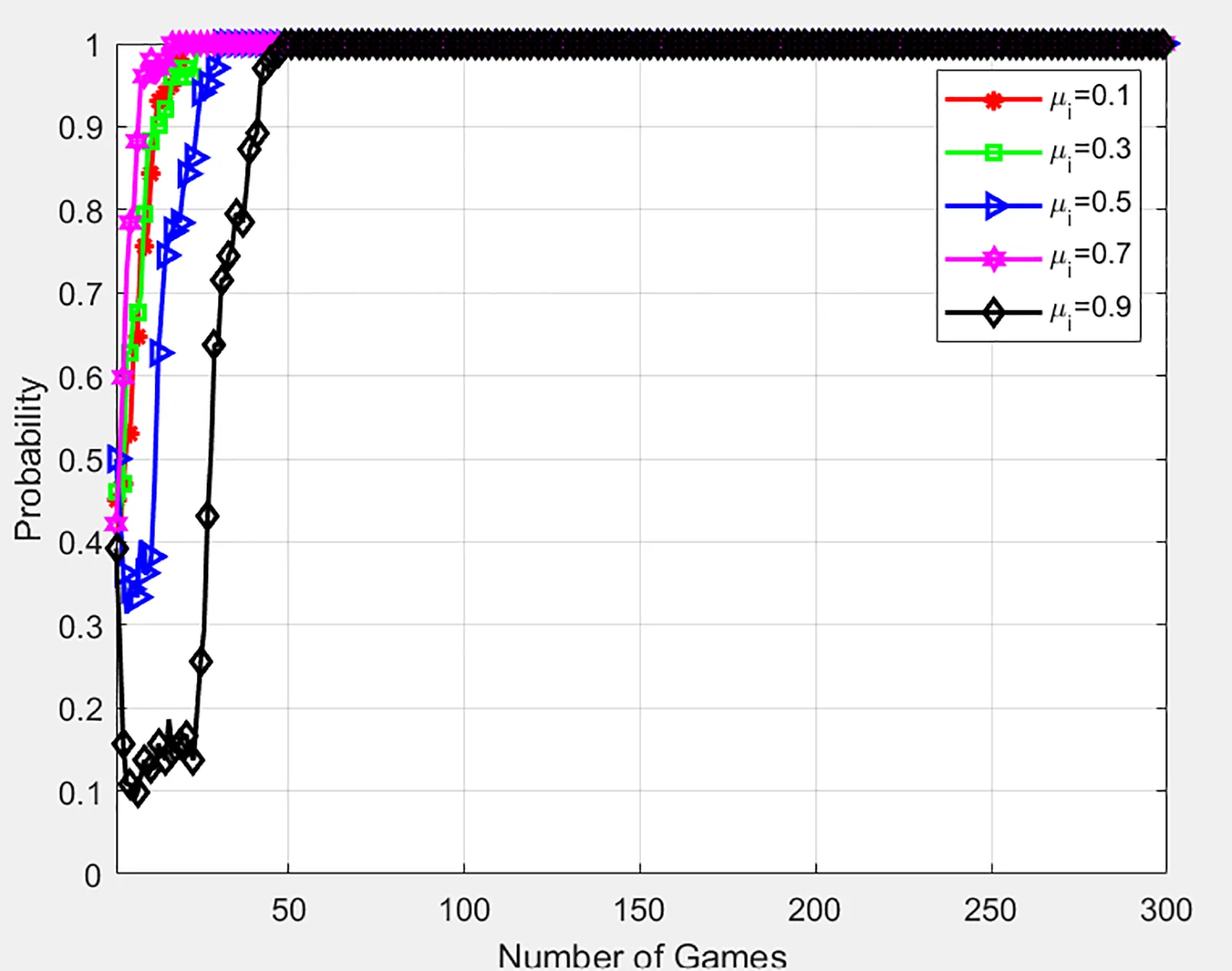
Impact of waste reduction spillover effect coefficient on network evolution at 100 nodes.

**Fig 19:**
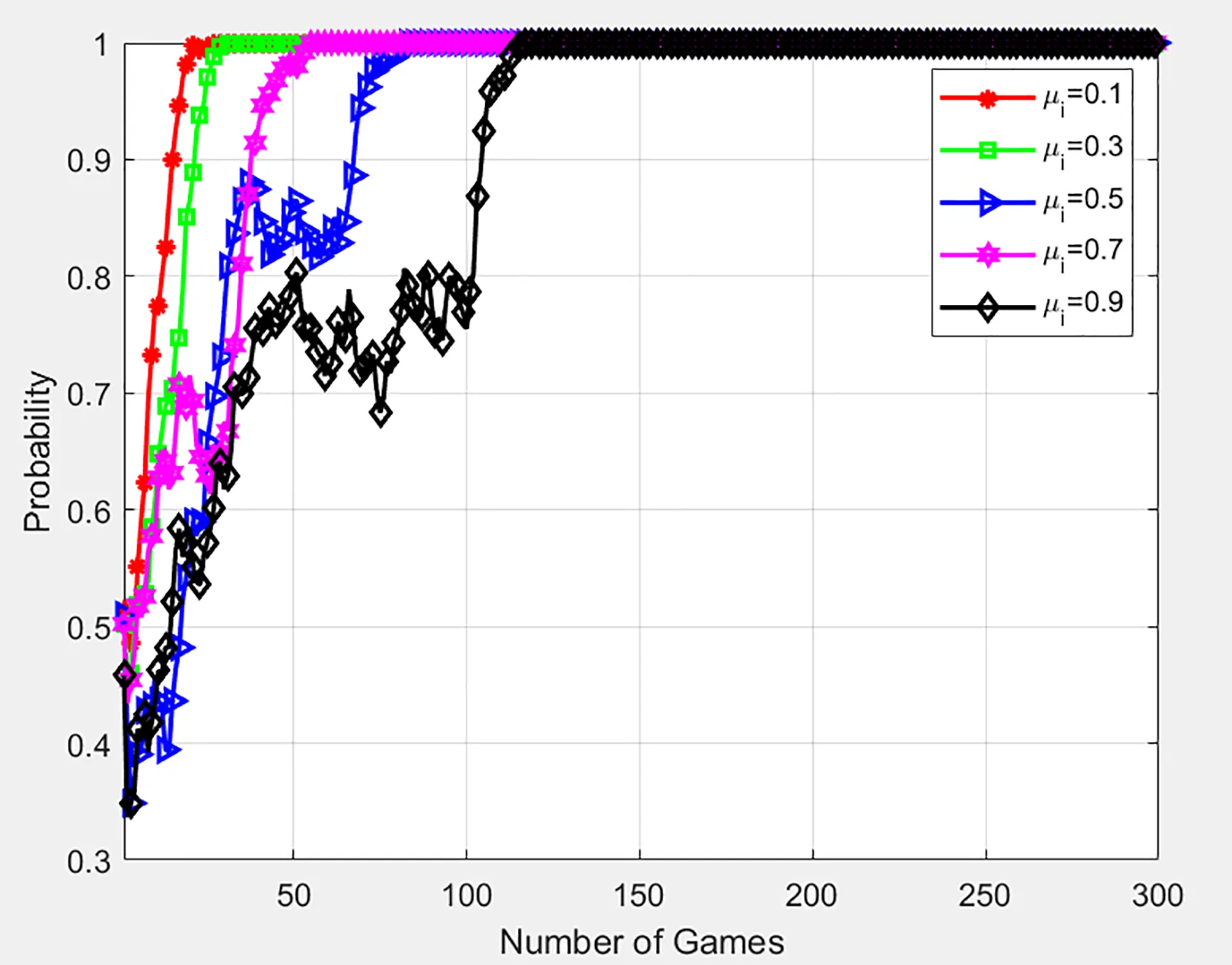
Impact of waste reduction spillover coefficients on network evolution at 500 node.

“Externalities” is an important feature of spillover effects, mainly referring to the welfare of a certain enterprise or subject to other enterprises or subjects, but is not reflected in the monetary or normal transaction [42]. Waste reduction behaviors among enterprises also show certain externalities, the node enterprises in the cooperative network carry out waste reduction activities, reduce the environmental pollution caused by waste emissions, and improve the utilization rate of resources, which inadvertently also brings certain benefits to other enterprises. The waste reduction spillover effect mentioned in this paper mainly refers to the assumption that one enterprise in the waste reduction network cooperation generates external benefits when it carries out waste reduction cooperation, and another enterprise obtains part of this subject’s waste reduction benefits without paying additional costs based on the sharing of waste reduction information and the complementarity of waste reduction knowledge. To study the coefficient of waste reduction spillover effect$\mu_{i}$ size on the evolution results of the cooperative network, the parameters other than the fixed waste reduction spillover effect coefficient are kept unchanged, and drawing on the practice of Shi in the study of the impact of spillover effect on the low-carbon cooperative behavior of the construction supply chain [39], the changes in the value of the waste reduction spillover effect coefficient are set as shown in Table 12, and the evolution of the three sizes of the cooperative network of 50 nodes, 100 nodes, and 500 nodes is discussed respectively. The specific evolution simulation results are shown in Figs 17 – 19.

**Table 12:** Parameterization of coefficients for waste reduction spillover effects of cooperative urban waste reduction behaviors.

network size	Spillover effects of waste reduction	network size	Spillover effects of waste reduction	network size	Spillover effects of waste reduction
50	0.1	100	0.1	500	0.1
50	0.3	100	0.3	500	0.3
50	0.5	100	0.5	500	0.5
50	0.7	100	0.7	500	0.7
50	0.9	100	0.9	500	0.9

From Fig 17 and Fig 18, it can be seen that in the cooperation networks with 50 and 100 nodes, within the range of parameter settings, the existence of the waste reduction spillover effect coefficient will make the waste reduction cooperation network eventually evolve and stabilize at 1. At this time, enterprises in the cooperation network are more inclined to choose the waste reduction cooperation strategy, which proves that the waste reduction spillover effect can promote waste reduction cooperation between enterprises. When the waste reduction spillover effect coefficient is set to 0.9, the evolution curve of the cooperation network tends to stabilize at a relatively slower speed and fluctuates more obviously in the early stage. Under moderate spillover effect coefficients, the cooperation probability rises faster and the network reaches the stable cooperative state earlier. This indicates that an appropriate waste reduction spillover effect is more conducive to the evolution of the cooperation network, while an excessively high spillover effect coefficient may slow down the evolution speed of the network.

In the evolution results of the waste reduction spillover effect coefficient in the 500-node network, it can be seen that the cooperation network eventually evolves and stabilizes at 1 under different spillover effect coefficients. However, compared with the 50-node and 100-node networks, the convergence speed of the 500-node network is relatively slower, and the fluctuation amplitude of the evolution curve is larger. Especially when the waste reduction spillover effect coefficient is 0.5 and 0.9, the evolution curve fluctuates more obviously in the early stage and needs a longer time to reach the stable cooperative state. Comparing the evolution results of the three different network scales, it can be seen that with the increase in the size of the cooperation network, the speed of network evolution slows down. This is mainly because the 500-node network has a larger scale, and the transmission of waste reduction cooperation information and strategy learning is slower, which may produce information asymmetry in the whole cooperation network and lead to larger fluctuations in the evolution curve.

In summary, in enterprise waste reduction network cooperation, the existence of the waste reduction spillover effect can promote cooperation between enterprises and the evolution of the cooperation network. However, with the increase of the waste reduction spillover effect coefficient, the evolution of the waste reduction cooperation network may slow down, especially in large-scale networks. An excessively high spillover effect coefficient may inhibit the efficient evolution of the network, although it does not necessarily change the final cooperative tendency. Therefore, to promote waste reduction cooperation between enterprises and accelerate the stable evolution of the cooperation network, the waste reduction spillover effect coefficient should be controlled within a reasonable range.

### 4.7 Related discussion and recommendations

From the analysis of the simulation results of the evolution game on the above four types of cooperation networks, it is concluded that in the process of the evolution of inter-firm waste reduction network cooperation, the different sizes of cooperation networks composed of different numbers of nodes, the cost-sharing coefficients, the penalty coefficients for breach of contract, the distribution coefficients of benefits, and the coefficients of the spillover effect of waste reduction will have an impact on the behavior of inter-firm cooperation on waste reduction. These factors will not only affect the final evolutionary tendency of the cooperation network, but also affect the evolution speed and stability of the cooperation network. Based on the simulation results of the cooperative network evolution, the following recommendations are proposed.

(1) Small-scale cooperation is more sensitive to changes in parameters, while large-scale cooperation is relatively less sensitive. Therefore, the Government should increase its support for central corporations (corporations with high nodal degrees) to promote the waste reduction effect of the whole cooperation network. Corporations should take the perspective of the overall cooperation network and combine their own advantages to cooperate with the government to promote the achievement of waste reduction goals.
(2) The setting of the cost-sharing coefficient affects the evolution of waste reduction cooperation; too large a coefficient may increase the cost burden of corporations and reduce their willingness to participate in cooperation, and may even cause corporations in small-scale networks to opt out of cooperation. In large-scale waste reduction networks, although the cooperation network may still evolve toward cooperation under some parameter settings, an excessive cost-sharing coefficient will slow down the evolution speed, increase fluctuations, and may keep the cooperation probability at a relatively low level for a long time. Therefore, the cost-sharing coefficient should be kept within a reasonable range to ensure the fairness and long-term stability of waste reduction cooperation. In large-scale waste reduction networks, corporations should be encouraged to participate in cooperation, which can reduce sensitivity to changes in the cost-sharing coefficient. The government can also support corporations in waste reduction technology research and development through subsidies to stimulate the motivation to participate in cooperation.
(3) Corporations should set up a fair and reasonable penalty mechanism to reduce “free-riding” behavior. A reasonable penalty coefficient can increase the cost of non-compliance, restrain opportunistic behavior, and promote corporations to choose the “cooperation” strategy. The government needs to strengthen the supervision of corporate cooperation to ensure that corporations fulfill their waste reduction tasks according to the contract and guarantee the effective operation of the whole cooperation network. At the same time, the penalty coefficient for non-compliance should be controlled within a reasonable range, especially in large-scale networks, so as to avoid over-indulgence or over-intervention in the waste reduction cooperation behavior of corporations.
(4) The benefit distribution coefficient is an important factor in promoting waste reduction cooperation. In a small-scale network, the benefit distribution coefficient has a greater impact on the stability of cooperation, and an excessively small benefit distribution coefficient may make the network evolve toward non-cooperation. When the benefit distribution coefficient is too small, corporations may not obtain sufficient benefits from cooperation and may choose to give up cooperation to protect their own interests. In larger-scale cooperation networks, unreasonable benefit distribution may slow down the evolution speed of the cooperation network and increase the difficulty of cooperation formation. Reasonable distribution coefficients can accelerate the stabilization of cooperative networks and promote the achievement of waste reduction goals. In cooperation networks of different sizes, corporations should formulate a reasonable distribution plan that meets the interests of all parties to ensure the long-term stability of cooperation.
(5) Waste reduction spillover effects can help promote cooperation among corporations, but an excessively high spillover effect may slow down the convergence speed and increase fluctuations in the evolution process, especially in large-scale networks. A certain waste reduction spillover effect can promote the diffusion of cooperative behavior among corporations and improve the overall waste reduction effect of the cooperation network. However, when the spillover effect coefficient is too large, the evolution curve may fluctuate more obviously and the network may need more time to reach a stable state. The government should support corporate innovation through R&D subsidies and other measures to promote broader waste reduction cooperation. At the same time, an effective communication mechanism should be established to encourage the sharing of experiences and achievements among corporations, thus improving the overall waste reduction effect.
(6) In large-scale waste reduction cooperation, there may be delays and asymmetries in the transmission of information, affecting the efficiency and stability of cooperation. To improve the efficiency of information transfer and reduce information asymmetry, the government should accelerate the construction of a smart waste management system to realize the supervision of the whole life cycle of waste and information sharing. This will not only help to improve the efficiency of cooperation among corporations, but also promote the construction of “waste-free cities”and enhance social and economic benefits.

## 5 Shortcomings and prospects

Based on combing the relevant literature on waste reduction cooperation at home and abroad, this paper analyzes in depth the waste reduction behaviors of the enterprises involved in waste reduction as well as the cooperative relationship among them, then constructs a cooperation model of urban waste reduction network, establishes an evolutionary game model on the cooperation network on this basis and carries out a stability analysis of the equilibrium point, and finally uses the MATLAB software carries out evolutionary simulation analysis on the behavior of network nodes to study the influence of each influencing factor parameter on the cooperative behavior of enterprises in waste reduction, and puts forward some targeted suggestions based on the simulation results.

There are some shortcomings in this paper that require further research and improvement.

(1) In the theoretical research part, the article assumes that waste reduction involves enterprises with limited rationality, which mainly stems from incomplete information and the ambiguity of organizational decision-making. In the future, if an extended rationality approach can be adopted to reduce the limited rationality of organizations, it will help to construct a model relatively closer to reality and put forward suggestions with more decision-making value.
(2) This paper constructs a variety of scale-free networks as a cooperative behavioral network model for corporate waste reduction, and in future research, scale-free networks and small-world networks can be combined to mine the behavioral characteristics between subjects, and further expand and enrich the structure of the cooperative behavioral network model between subjects.
(3) In constructing a game model, a node in the cooperative network and its neighboring nodes as a representative of the game research has a certain degree of representativeness but does not fully represent the entire cooperative network of the game, because of the cooperative network of nodes involved in more, and each node in the status of the entire cooperative network is not identical, there are a number of nodes degree of large enterprises. If the status of nodes is included in the threshold of further exploration, it will certainly outline a relatively more objective and decision-support value of the urban waste reduction network cooperation picture.

## Supporting information

S1 File Supplementary Appendix, Data and Code. **S1 Appendix A.** Stability assessment of the complete 2000-round simulations. This file contains Appendix A and Table A1. Table A1 summarizes the final cooperation probability, the average cooperation probability over the last 100 rounds, the maximum standard deviation over the last 100 rounds, and the convergence round range for Figs 8–19 based on the complete 2000-round simulation results. **S2 Data.** Simulation output data underlying Figs 8–19 and S1A Appendix. This file contains the complete 2000-round simulation output data underlying Figs 8–19 and S1A Appendix, including trajectory data, stability assessment results, and curve-level results in CSV format. It also includes the original MATLAB  .mat output file as an additional raw simulation data file. **S3 Code.** MATLAB simulation code used for the evolutionary game simulations. This file contains the final MATLAB scripts used to generate the evolutionary simulation results shown in Figs 8–19. Random seeds were fixed in the scripts to ensure reproducibility of the simulation results.
(ZIP)
