# Supplementary material for: The cooperative behavioral game of urban waste reduction network considering environmental cost-sharing
Source: PLoS One. 2026 Sep 1;21(9):e0356910. doi: 10.1371/journal.pone.0356910 (PMC13533397; doi:10.1371/journal.pone.0356910)
Supplement: Supplementary file 1 [file pone.0356910.s001.zip › Supporting Information/S1_Appendix A.docx]

# Appendix A. Stability assessment of the complete 2000-round simulations

All evolutionary games were run for 2000 rounds. For readability, Figs. 8–19 present only the first 300 rounds. The convergence state and stability of each trajectory were evaluated using the complete 2000-round simulation results. Table A1 summarizes the final cooperation probability, the average cooperation probability over the last 100 rounds, and the convergence round range for each figure.

**Table A1. Summary stability assessment based on the complete 2000-round simulation results**

| **Fig.** | **Factor** | **Nodes** | **Final PtC range** | **Last-100 avg range** | **Max last-100 SD** | **Convergence round range** | **Stability** |
| --- | --- | --- | --- | --- | --- | --- | --- |
| Fig. 8 | Cost-sharing | 50 | 0.000-1.000 | 0.000-1.000 | 0.000 | 11-120 | Stable; mixed states |
| Fig. 9 | Cost-sharing | 100 | 1.000 | 1.000 | 0.000 | 29-126 | Stable; all cooperate |
| Fig. 10 | Cost-sharing | 500 | 0.273-1.000 | 0.270-1.000 | 0.009 | 22-1994 | Stable; mixed/partial states |
| Fig. 11 | Default penalty | 50 | 1.000 | 1.000 | 0.000 | 10-34 | Stable; all cooperate |
| Fig. 12 | Default penalty | 100 | 1.000 | 1.000 | 0.000 | 11-29 | Stable; all cooperate |
| Fig. 13 | Default penalty | 500 | 1.000 | 1.000 | 0.000 | 22-64 | Stable; all cooperate |
| Fig. 14 | Benefit distribution | 50 | 0.000-1.000 | 0.000-1.000 | 0.000 | 5-21 | Stable; mixed states |
| Fig. 15 | Benefit distribution | 100 | 1.000 | 1.000 | 0.000 | 8-71 | Stable; all cooperate |
| Fig. 16 | Benefit distribution | 500 | 1.000 | 1.000 | 0.000 | 27-75 | Stable; all cooperate |
| Fig. 17 | Spillover effect | 50 | 1.000 | 1.000 | 0.000 | 11-38 | Stable; all cooperate |
| Fig. 18 | Spillover effect | 100 | 1.000 | 1.000 | 0.000 | 16-47 | Stable; all cooperate |
| Fig. 19 | Spillover effect | 500 | 1.000 | 1.000 | 0.000 | 21-114 | Stable; all cooperate |

Note: Final PtC range reports the range of final cooperation probabilities across the five tested values of the corresponding coefficient in each figure. A range of 0.000–1.000 indicates that different parameter settings converge to different stable states, rather than that a single trajectory remains unstable.

Stable; all cooperate = all five parameter trajectories converge to PtC = 1. Stable; mixed states = parameter trajectories converge to both cooperative and non-cooperative stable states. Stable; mixed/partial states = the trajectories include cooperative and intermediate low-cooperation stable states.

The convergence round is defined as the earliest round after which the cooperation probability remains within 0.01 of its final value until the end of the 2000-round simulation
